# *In silico* analysis of the tryptophan hydroxylase 2 (TPH2) protein variants related to psychiatric disorders

**DOI:** 10.1371/journal.pone.0229730

**Published:** 2020-03-02

**Authors:** Gabriel Rodrigues Coutinho Pereira, Gustavo Duarte Bocayuva Tavares, Marta Costa de Freitas, Joelma Freire De Mesquita

**Affiliations:** Bioinformatics and Computational Biology Laboratory, Department of Genetics and Molecular Biology, Federal University of the State of Rio de Janeiro (UNIRIO), Rio de Janeiro, Rio de Janeiro, Brazil; University of Akron, UNITED STATES

## Abstract

The tryptophan hydroxylase 2 (TPH2) enzyme catalyzes the first step of serotonin biosynthesis. Serotonin is known for its role in several homeostatic systems related to sleep, mood, and food intake. As the reaction catalyzed by TPH2 is the rate-limiting step of serotonin biosynthesis, mutations in TPH2 have been associated with several psychiatric disorders (PD). This work undertakes an *in silico* analysis of the effects of genetic mutations in the human TPH2 protein. Ten algorithms were used to predict the functional and stability effects of the TPH2 mutations. ConSurf was used to estimate the evolutionary conservation of TPH2 amino acids. GROMACS was used to perform molecular dynamics (MD) simulations of TPH2 WT and P260S, R303W, and R441H, which had already been associated with the development of PD. Forty-six TPH2 variants were compiled from the literature. Among the analyzed variants, those occurring at the catalytic domain were shown to be more damaging to protein structure and function. The ConSurf analysis indicated that the mutations affecting the catalytic domain were also more conserved throughout evolution. The variants S364K and S383F were predicted to be deleterious by all the functional algorithms used and occurred at conserved positions, suggesting that they might be deleterious. The MD analyses indicate that the mutations P206S, R303W, and R441H affect TPH2 flexibility and essential mobility at the catalytic and oligomerization domains. The variants P206S, R303W, and R441H also exhibited alterations in dimer binding affinity and stability throughout the simulations. Thus, these mutations may impair TPH2 functional interactions and, consequently, its function, leading to the development of PD. Furthermore, we developed a database, SNPMOL (http://www.snpmol.org/), containing the results presented in this paper. Understanding the effects of TPH2 mutations on protein structure and function may lead to improvements in existing treatments for PD and facilitate the design of further experiments.

## Introduction

Psychiatric disorders (PD) are considered a major public health problem worldwide [1]. In the United States and Europe, the estimated prevalence of PD is approximately 25 to 30% [2]. The high disability of PD severely impacts the patient’s social and professional environment, resulting in loss of income and productivity, especially due to the patient’s absence from work and early retirement. The estimated cost of PD is comparable to that of cancer, diabetes, and chronic respiratory diseases combined [1]. Despite the relevance of PD, most of the drugs available for their treatment have shown several adverse effects, such as insomnia and anxiety, leading to a loss in quality of life [3,4].

The tryptophan hydroxylase 2 (TPH2) protein belongs to a family of iron and tetrahydrobiopterin-dependent aromatic amino acid hydroxylases [5,6]. The structure of TPH2 is composed of three functional domains: a regulatory NH_2_-terminal domain, a central catalytic domain, and a COOH-terminal oligomerization domain [7,8]. TPH2 is an enzyme that catalyzes the first and rate-limiting reaction of serotonin (5-HTP) biosynthesis in neuronal and enteric serotonergic cells, by hydroxylating L-tryptophan in 5-hydroxytryptophan (5-HTP) [6,7]. Serotonin is an important neurotransmitter that regulates a wide range of physiological processes related to the central nervous system, such as sleeping and mood [9]. Alterations in serotonin levels and central serotonergic system deregulation cause brain dysfunction, ultimately leading to the development of PD [10]. Mutations in the *TPH2* gene are known to affect the structure and function of the TPH2 protein, resulting in reduced serotonin synthesis [8,11]. These mutations have been previously associated with bipolar affective disorder [12], attention-deficit/hyperactivity disorder [8], and major depression [13].

Computer simulations can perform predictions with a variety of purposes, such as predicting the effect of mutations on proteins and modeling their three-dimensional structures in a better cost/efficiency, faster butaccurate manner [14]. Thus, this method has become an effective approach for studying disease‐related mutations and their implications for protein structure and function [15]. Thus, in this work, we applied computer simulations to the study of TPH2 protein variants, aiming to characterize their effects on protein structure to better understand their molecular mechanism of pathology [16]. Since these variants can affect protein structure and, consequently, protein-drug binding [17], knowing their effect on protein structure may also help to understand their impact on drug selection, dosing, and adverse effects [18]. The results of this study also favor the design of further experiments [15] and provide relevant information that could lead to improvements in PD treatments, especially in the field of precision medicine [17].

## Materials and methods

### Dataset

The experimental structure of wild-type (WT) TPH2 was obtained from the Protein Data Bank (PDB) database [19], and its sequence was obtained from the UniProt database. The TPH2 variants were compiled from the UniProt, OMIM, dbSNP [15,20] and PubMed database.

### Functional and stability prediction

Functional and structural analyses of the TPH2 variants were predicted using ten different algorithms: I-Mutant, Mutpred, PhD-SNP, nsSNP-Analyzer, Pmut, SNP&GO, SNAP2, Polyphen-2, SNPEffect [15], and VarMod [21].

### Evolutionary conservation analysis

The evolutionary conservation score of each amino acid of TPH2 was determined using the ConSurf algorithm, based on the phylogenetic relationship of its sequence and homologous sequences [22]. The following parameters were selected for this analysis: homologous search algorithm: CSI-BLAST; protein database: UNIREF-90; alignment method: Bayesian; calculation method: MAFFT-L-INS-i; evolutionary substitution model: best model; number of iterations: 3; E-value cut-off: 0.0001; number of reference sequences selected: 150; maximum sequence identity: 95%; minimum identity for counterparts: 35%.

### Molecular dynamics simulations

The molecular dynamics (MD) simulations of TPH2 WT and its variants P206S, R303W, and R441H were performed using the GROMACS 5.0.7 package. The software Visual Molecular Dynamics 1.9.2 was used to induce the mutations P206S, R303W, and R441H on the crystallographic structure of TPH2 WT [15]. The mutations P206S, R303W, and R441H were selected for the MD simulations because they have already been associated with the development of PD [11].

The MD simulations were performed according to the methodology described by Pereira et al., 2019. AMBER99SB-ILDN was selected as the force field of the simulations. The MD preparation consisted of adding TIP3P water models inside a cubic box. The systems were then neutralized by adding Na^+^ and Cl^-^ ions and minimized for 5000 steps using the *steepest descent* method. After system minimization, NVT and NPT ensembles were performed for 100 psec at a pressure of 1 atm and temperature of 300 K. V-rescale was selected as the thermostat, and Parrinello-Rahman was selected as the barostat of the NVT and NPT ensembles. The production simulations were performed at 300 K for a duration of 100 nsec using the LINCS (linear constraint solver) and PME (particle mesh Ewald) algorithms.

The MD trajectories were analyzed using the following GROMACS distribution programs: *gmx rms*, *gmx rmsf*, *gmx gyrate*, *gmx dssp*, *gmx sasa*, and *gmx hbond*. To perform principal component analysis (PCA) for WT TPH2 and its variants, the MD trajectories were also analyzed using the Bio3D library implemented in R software [23]. Bio3D was used to perform principal component analysis (PCA) for the WT TPH2 and its variants. PCA was performed for the Cα atoms, and their Cartesian coordinates were used to generate the covariance matrices. Rotational and translational motions were also removed for the construction of the covariance matrices. MM-PBSA analysis of WT TPH2 and its variants was performed using the *g_mmpbsa* software implemented in GROMACS [24].

## Results

### Dataset

**Fig 1:**
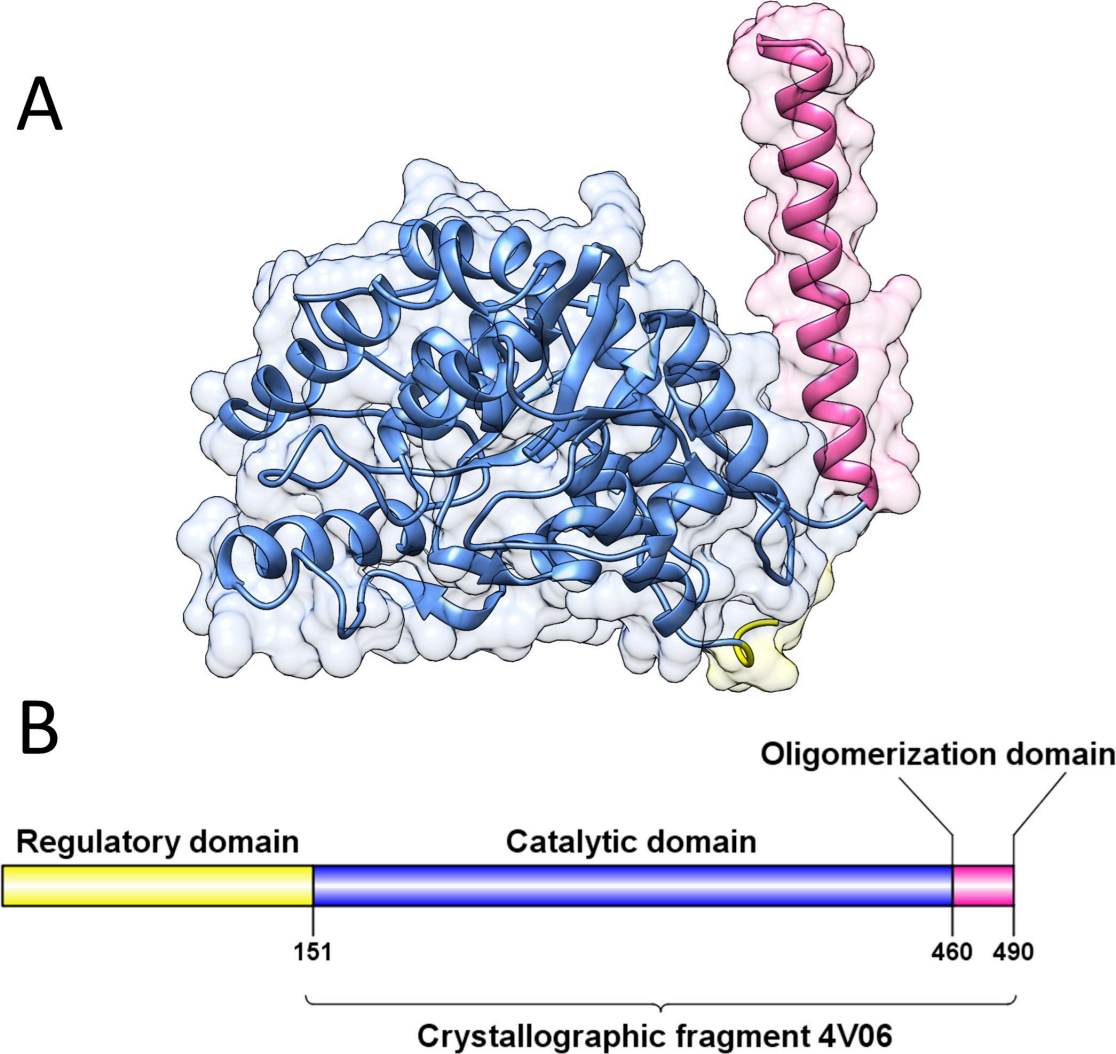
Crystallographic structure and schematic representation of TPH2. The regulatory, catalytic and oligomerization domains of TPH2 are represented in yellow, blue and pink, respectively. (A) Secondary structure representation of the crystallographic structure of TPH2 (PDB ID: 4V06). The protein surface is also shown. (B) Schematic representation of TPH2. The region corresponding to the experimentally determined structure of TPH2 is indicated by the bracket.

The crystallographic structure of TPH2 retrieved from the Protein Data Bank [PDB ID: 4v06] is a 342-amino acid fragment (Fig 1A). As shown in Fig 1B, the crystallographic fragment comprises residues 148 to 490 of TPH2, which includes its catalytic domain (residues 151–459) and oligomerization domain (residues 460–490) [6]. No structure has already been experimentally determined for the regulatory domain of TPH2 [11].

The complete amino acid sequence of the TPH2 protein was retrieved from the UniProt database [UniProt ID: Q8IWU9]. Forty-six mutations were found in the TPH2 sequence: six at the oligomerization domain, 21 at the regulatory domain and 19 at the catalytic domain.

### Functional and stability prediction

**Fig 2:**
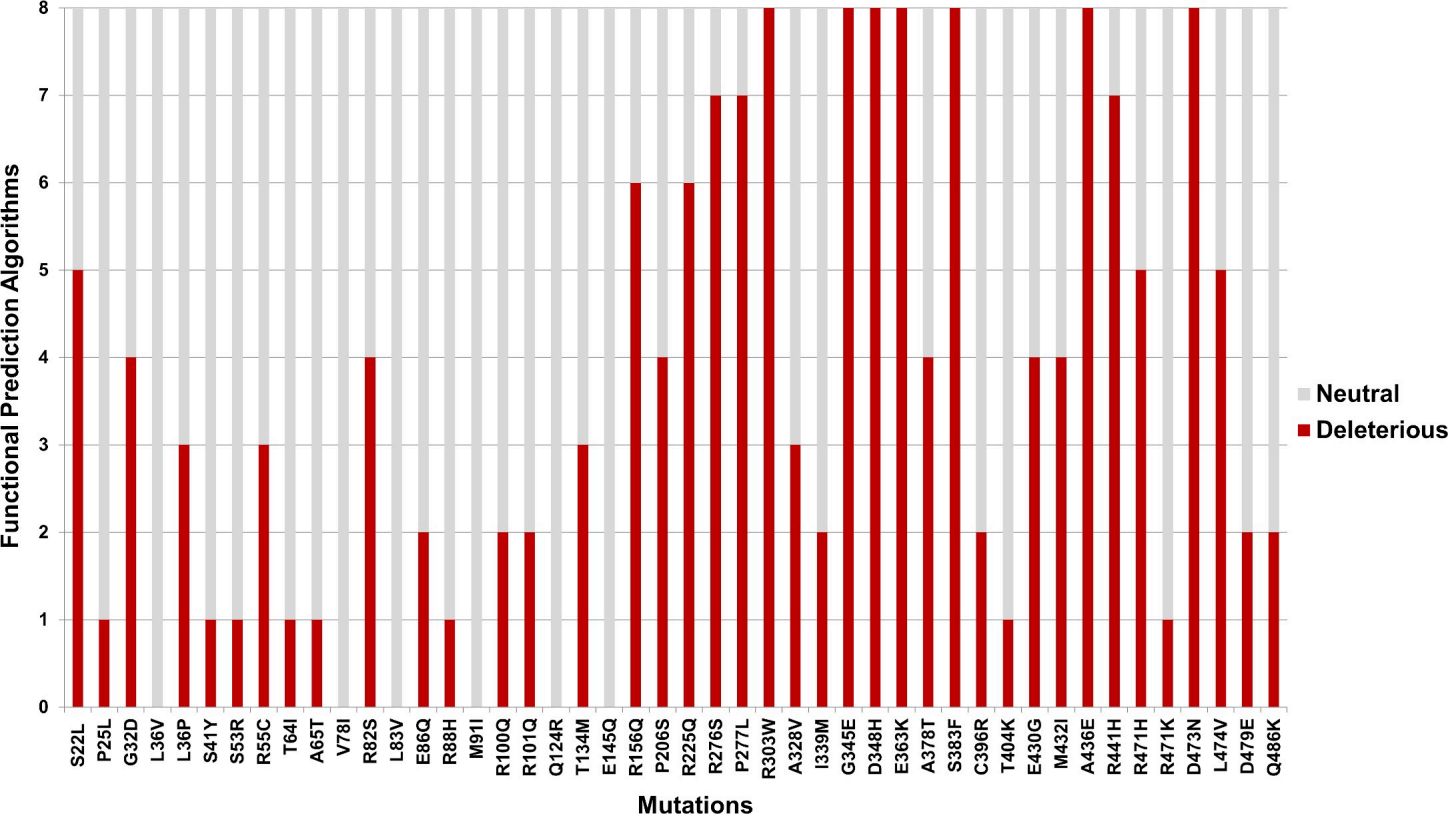
Functional prediction of TPH2 mutations. The 46 known TPH2 mutations were analyzed using eight different functional prediction algorithms. The bar plot indicates the number of neutral (gray bars) and deleterious (red bars) predictions for each TPH2 mutation.

SNAP2, VarMod, PhD-SNP, SNP&GO, I-Mutant Disease, nsSNPAnalyzer, Pmut, and MutPred were used to predict the functional effects of the TPH2 variants (S1 Table). The variants P206S, R303W, and R441H, which have been previously associated with the development of PD [11], were predicted as deleterious by 44%, 100% and 89% of the algorithms (Fig 2), respectively. Moreover, the variants G345E, E363K, and S383F were predicted to be deleterious by 100% of the algorithms (Fig 2), while the variants V78I, L83V, M91I, Q124R, and E145Q were predicted to be neutral for 100% of the algorithms used. This analysis also showed that more than 50% of the functional prediction algorithms classified the TPH2 mutations at the regulatory domain as neutral, while most of them classified the mutations at the catalytic domain as deleterious (Fig 2).

The effects of TPH2 mutations on chaperone binding (LIMBO), amyloid propensity (WALTZ), and protein aggregation (TANGO) were predicted using the SNPEffect algorithm. According to the SNPEffect prediction (S2 Table), the variants T64I, R225Q, S383F, and R441H increase protein aggregation; R82S and M91I increase amyloid propensity; and R225Q increases chaperone binding. The other TPH2 variants showed no effects on these features.

Furthermore, the effects of the TPH2 variants on protein stability were predicted using the I-Mutant and FoldX algorithms (S3 Table). The FoldX algorithm was unable to predict the mutations that occurred at the regulatory domain of TPH2 since no structure has been experimentally determined for this region [18]. According to the I-Mutant and FoldX predictions, the variants P206S, R276S, P277L, R430G, M432I, A436E, R441H, R471H, and L474V reduce protein stability, while the variants R383F, D479E, and Q486K do not affect protein stability. The other TPH2 variants, including R303W, were predicted to affect protein stability by at least one algorithm. However, none of them was predicted to increase protein stability by the I-Mutant and FoldX algorithms at the same time.

### Evolutionary conservation analysis

**Fig 3:**
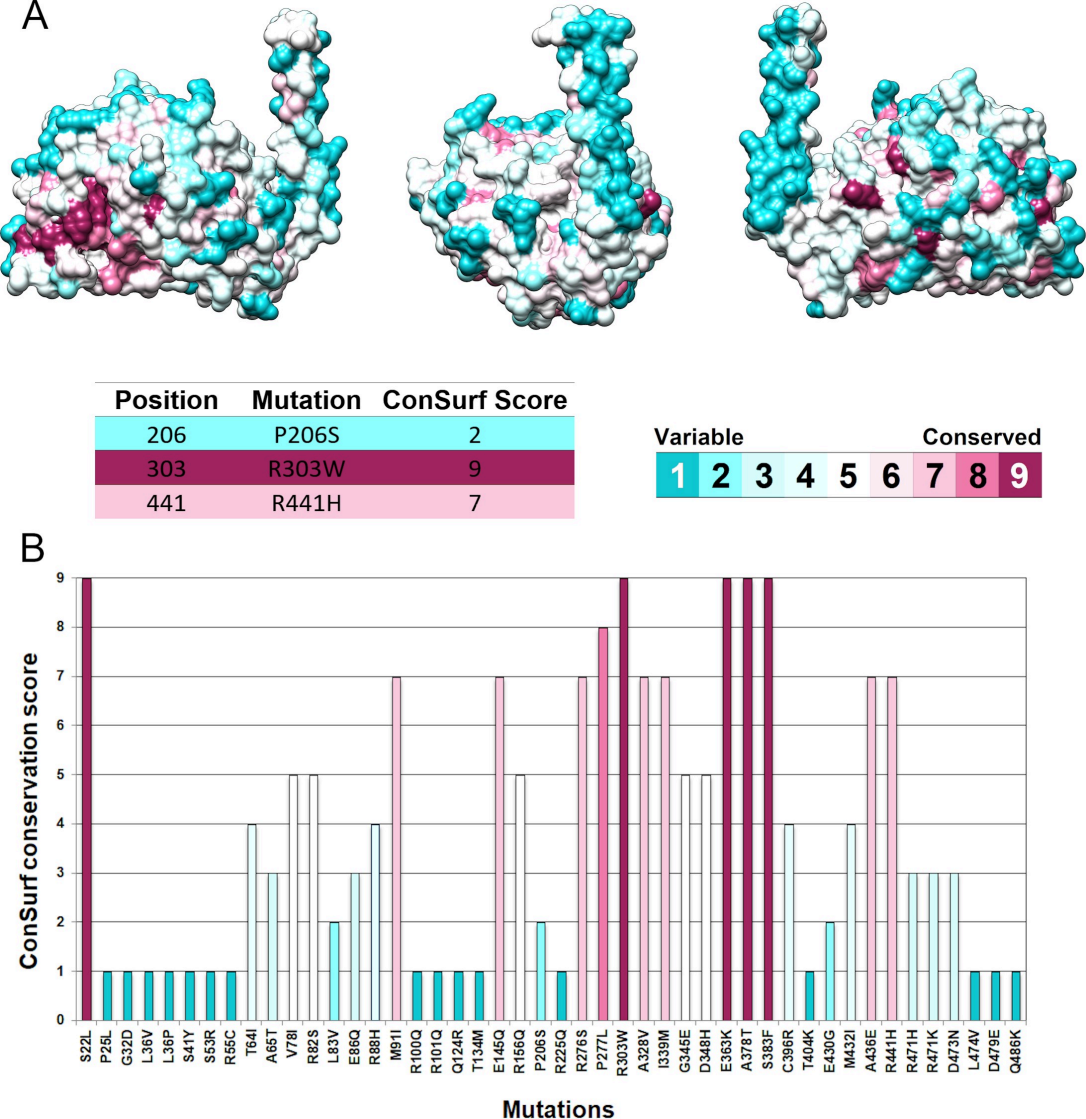
Evolutionary conservation analysis of TPH2 protein. (A) The evolutionary conservation profile of the TPH2 protein is shown at three different angles. Each amino acid of TPH2 is represented as a space-filling model and colored according to its ConSurf conservation score. The color-coding bar shows the ConSurf coloring scheme, which varies from cyan and highly variable to maroon and highly conserved. The conservation score for each amino acid of TPH2 affected by deleterious mutations, i.e., P206S, R303W, and R441H, are also shown. (B) The bar plot shows the ConSurf score for each amino acid of TPH2 affected by mutations. The bar plot was colored according to the ConSurf coloring scheme.

The crystallographic structure of TPH2 was submitted to the ConSurf server for assessing its evolutionary conservation. ConSurf estimated the evolutionary conservation of TPH2 residues and attributed to them conservation scores, which were projected on the protein’s surface [22]. The ConSurf conservation score is calculated by comparing a given amino acid sequence to its homologous sequences [25]. The THP2 residues were colored according to their conservation scores, ranging from blue and variable to maroon and conserved (Fig 3).

According to the ConSurf analysis (Fig 3), positions R303 and R441 of TPH2 were classified as highly conserved, while position P206 was classified as variable. Moreover, the mutations S22L, E363K, A378T, and S383F occur at highly conserved positions. This analysis also showed that mutations in the TPH2 regulatory domain occur mostly in variable residues, while mutations in the catalytic domain occur mostly in conserved residues.

### Molecular dynamics simulations

The MD simulation is an *in silico* method of solving Newtonian equations of motion for a group of atoms [26]. This method describes the variation of molecular movement over time and can be used to reproduce the behavior of proteins in their biological environment [14]. During an MD simulation, the interatomic interactions of a given molecular system are calculated over time. Their corresponding atomic coordinates are recorded at each simulation step, generating a trajectory file that provides detailed information on changes in protein conformation and fluctuation that can be used to assess structural parameters [15].

MD simulations of TPH2 WT and its variants P206S, R303W, and R441H were performed to better understand the impact of these amino acid substitutions on the TPH2 structure. The following structural parameters were analyzed from the MD trajectories: root-mean-square deviation (RMSD), root-mean-square fluctuation (RMSF), B-factor, radius of gyration (Rg), solvent accessible surface area (SASA), secondary structure (SS), principal components (PC), hydrogen bonds (Hb) and hydrophobic contacts. Binding free energy and their decomposed van der Waals (vdW) and electrostatic interactions were also analyzed.

**Fig 4:**
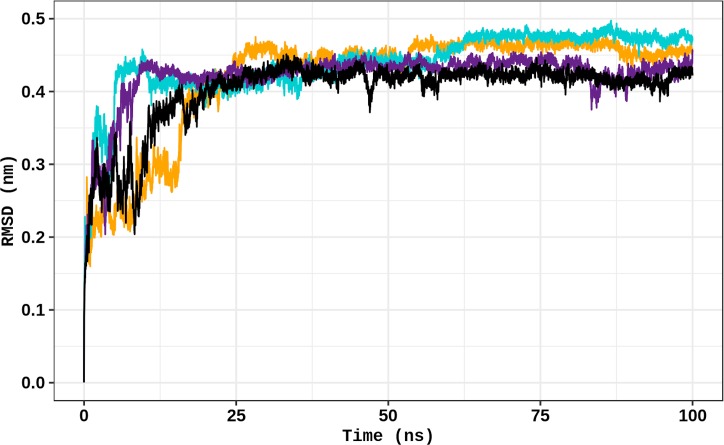
Backbone RMSD of WT TPH2 and its variants. The RMSD for the backbone atoms of TPH2 WT and variants at 300 K is shown as a function of time. WT is represented in black, variant P206S is represented in dark yellow, variant R303W is represented in purple, and variant R441H is represented in turquoise.

RMSD is a measure of the spatial differences between a starting structure and its corresponding coordinates computed during the simulation. This parameter is therefore useful to analyze the time-dependent motion of a given structure and to determine its structural convergence throughout the simulation [15]. The RMSD was calculated from the total number of conformations computed during the 100 ns simulations (Fig 4). A sudden increase in the RMSD values of all simulations was observed at the beginning of the simulations. The establishment of a plateau in the RMSD values indicates that the protein structures fluctuate around stable average conformations [25]. The WT, P206S, and R303W structures start to float to a stable conformation first, at 30 ns followed by R441H at approximately 60 ns. The initial effects of the trajectories were then discounted for further analyses.

**Fig 5:**
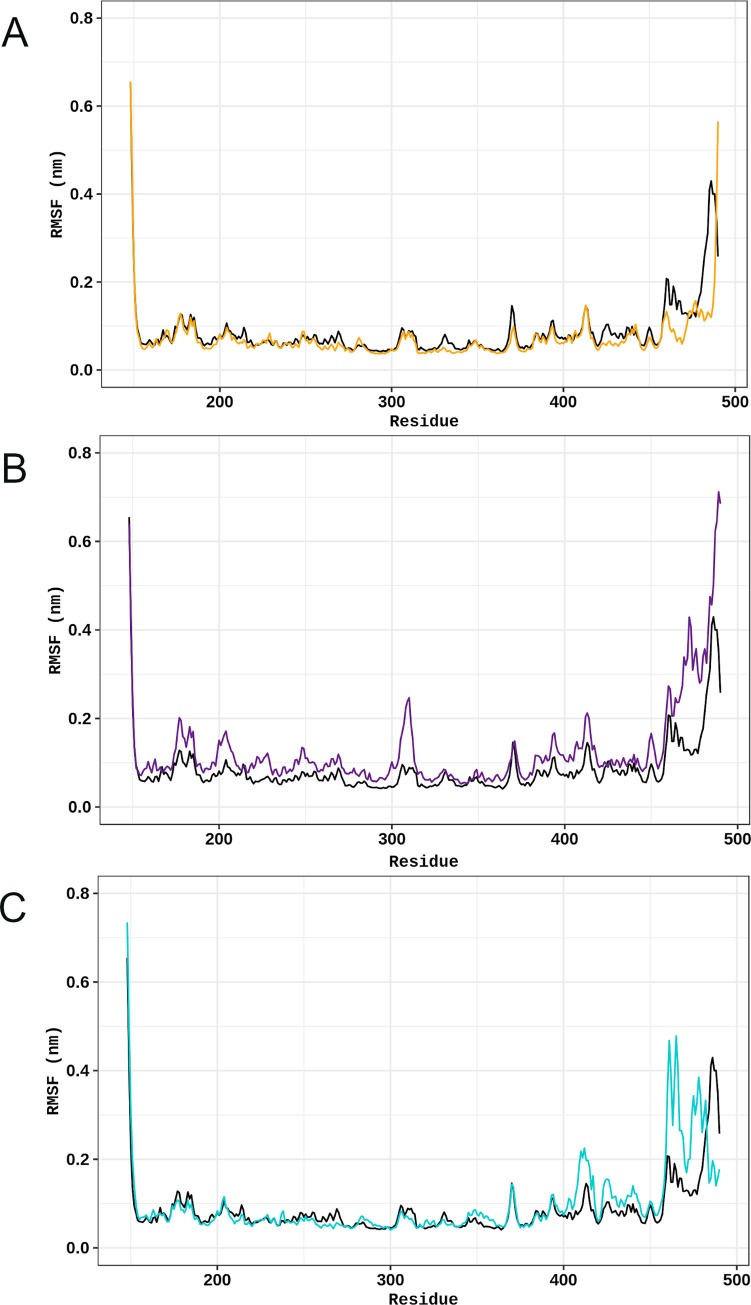
RMSF per residue of WT TPH2 and its variants. The RMSF of each residue of TPH2 WT and variants at 300 K is shown as a line plot. (A) Comparison between the WT (black) and variant P206S (dark yellow). (B) Comparison between the WT (black) and variant R303W (purple). (C) Comparison between the WT (black) and variant R441H (turquoise).

RMSF is a measure of the structural displacement of a given amino acid from its corresponding average position throughout the simulation. This parameter is then useful to describe the local flexibility, allowing the identification of rigid and flexible regions in proteins [15]. The RMSF of the TPH2 variants was analyzed and compared to the WT. The RMSF analysis (Fig 5) pointed to flexibility alterations at the oligomerization domain of all analyzed TPH2 variants. This analysis also pointed to increased flexibility at the terminal portion of the catalytic domain of the R303W and R441H variants, as well as increased flexibility near the mutated site of the R303W variant. Thus, this finding suggested flexibility alterations in all analyzed variants.

**Fig 6:**
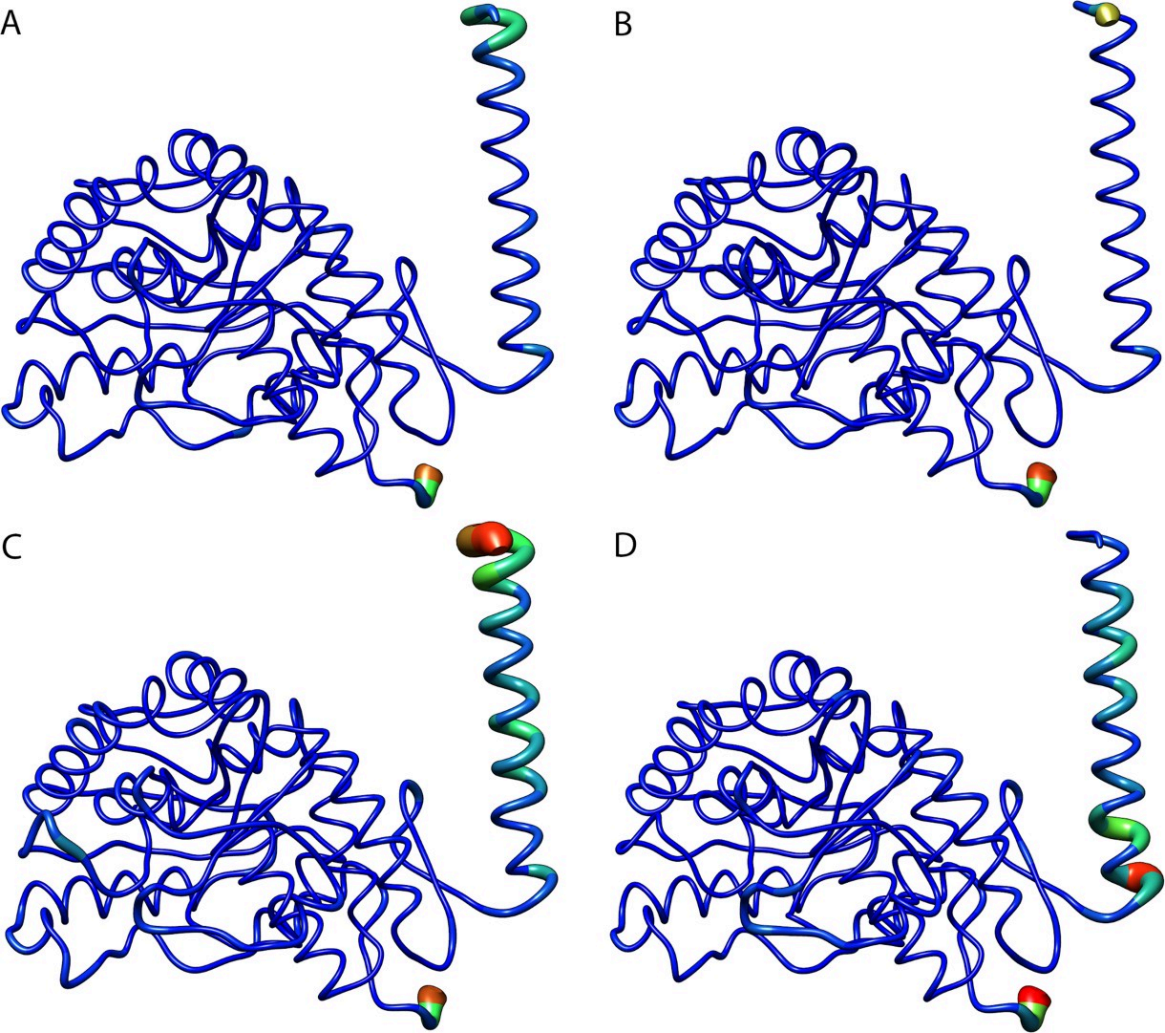
B-factor per residue of WT TPH2 and its variants. Each residue of TPH WT (A) and variants P206S (B), R303W (C), and R441H (D) is colored and sized according to its B-factor. The coloring-thickness scale varies from dark blue and thin (highly rigid residues) to red and thick (highly flexible residues).

The B-factor, also known as a temperature-displacement factor, is a measure of the structural displacement of a given amino acid due to thermal vibrations. The B-factor is then useful to assess the structural flexibility throughout the simulation [25]. This analysis also provides an interesting three-dimensional representation of structural flexibility. The B-factor values for each amino acid of TPH2 WT and its variants were projected on the corresponding protein surface following a coloring-thickness scheme that varies from blue and thin (rigid residues) to red and thick (flexible residues) (Fig 6). The B-factors of the TPH2 variants were analyzed and compared to the WT. The B-factor analysis also pointed to flexibility alterations at the oligomerization domain (C-terminal alpha-helix) of all analyzed variants. The flexibility alterations are particularly high at the oligomerization domain of R303W and R441H variants. The B-factor analysis also pointed to little flexibility alterations at the catalytic domain of the R303W and R441H variants. The B-factor analysis thus suggested flexibility changes in all TPH2 variants at regions similar to those found altered in the RMSF analysis.

PCA is also known as essential dynamics (ED) when it is used to analyze MD simulations. This statistical technique extracts the correlated structural motions responsible for the largest variance (essential motions) in a molecular trajectory using a covariance matrix constructed from the atomic positions (Cartesian coordinates) [27]. Generally, the first eigenvectors of the covariance matrix, also called PCs, successfully describe almost all essential subsets of protein conformations [28]. Thus, the projection of a protein trajectory on the first PCs is useful to describe its essential dynamics [25].

**Fig 7:**
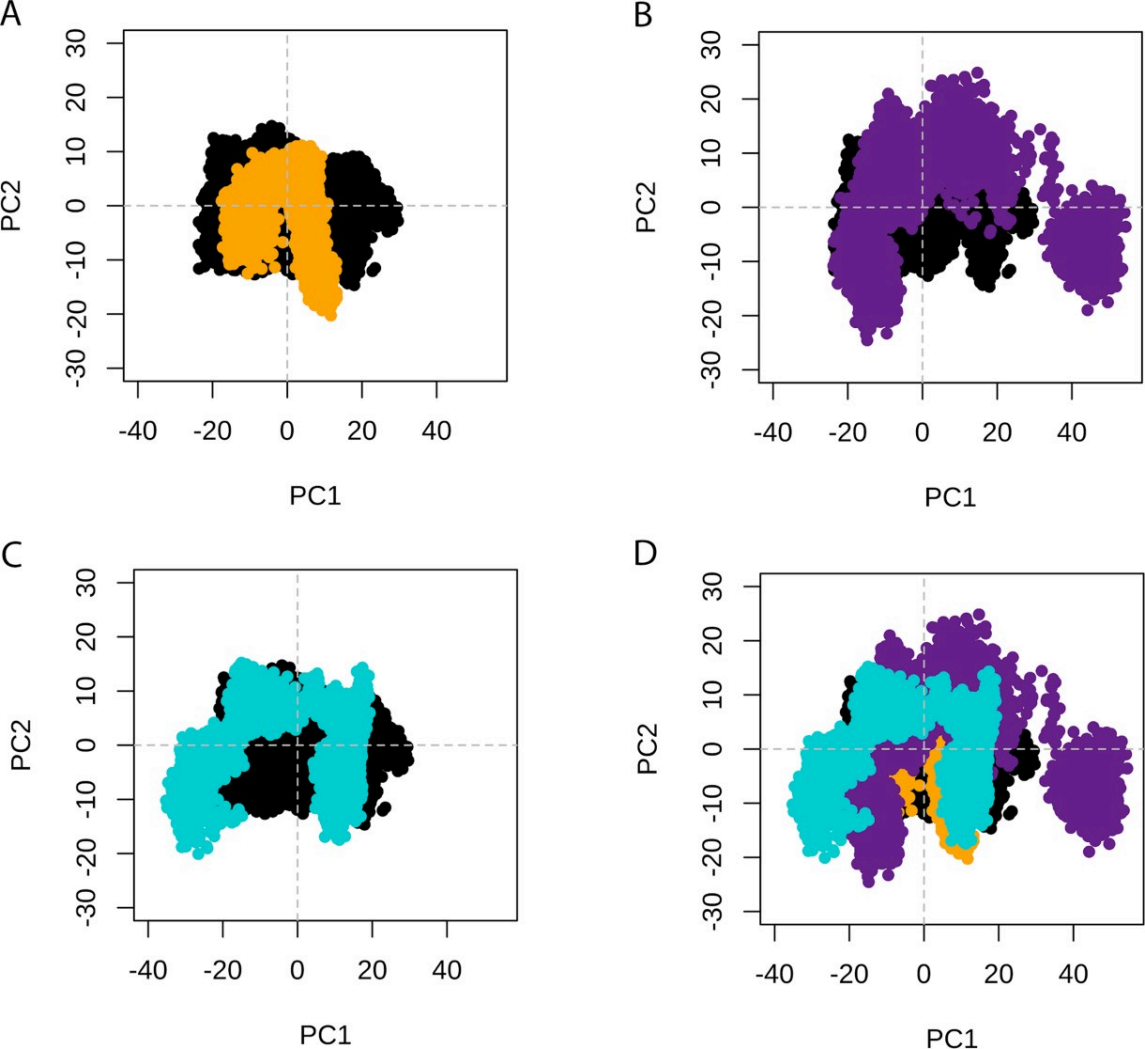
PCA of WT TPH2 and its variants. Projection of the first two principal components (PC1 and PC2) extracted from the essential dynamics. (A) PCA comparison between WT TPH2 (black) and its variant P206S (dark yellow). (B) PCA comparison between WT TPH2 (black) and its variant R303W (purple). (C) PCA comparison between WT TPH2 (black) and its variant R441H (turquoise). (D) Superimposed PCA comparison between WT TPH (black) and its variants P206S (dark yellow), R303W (purple), and R441H (turquoise).

**Fig 8:**
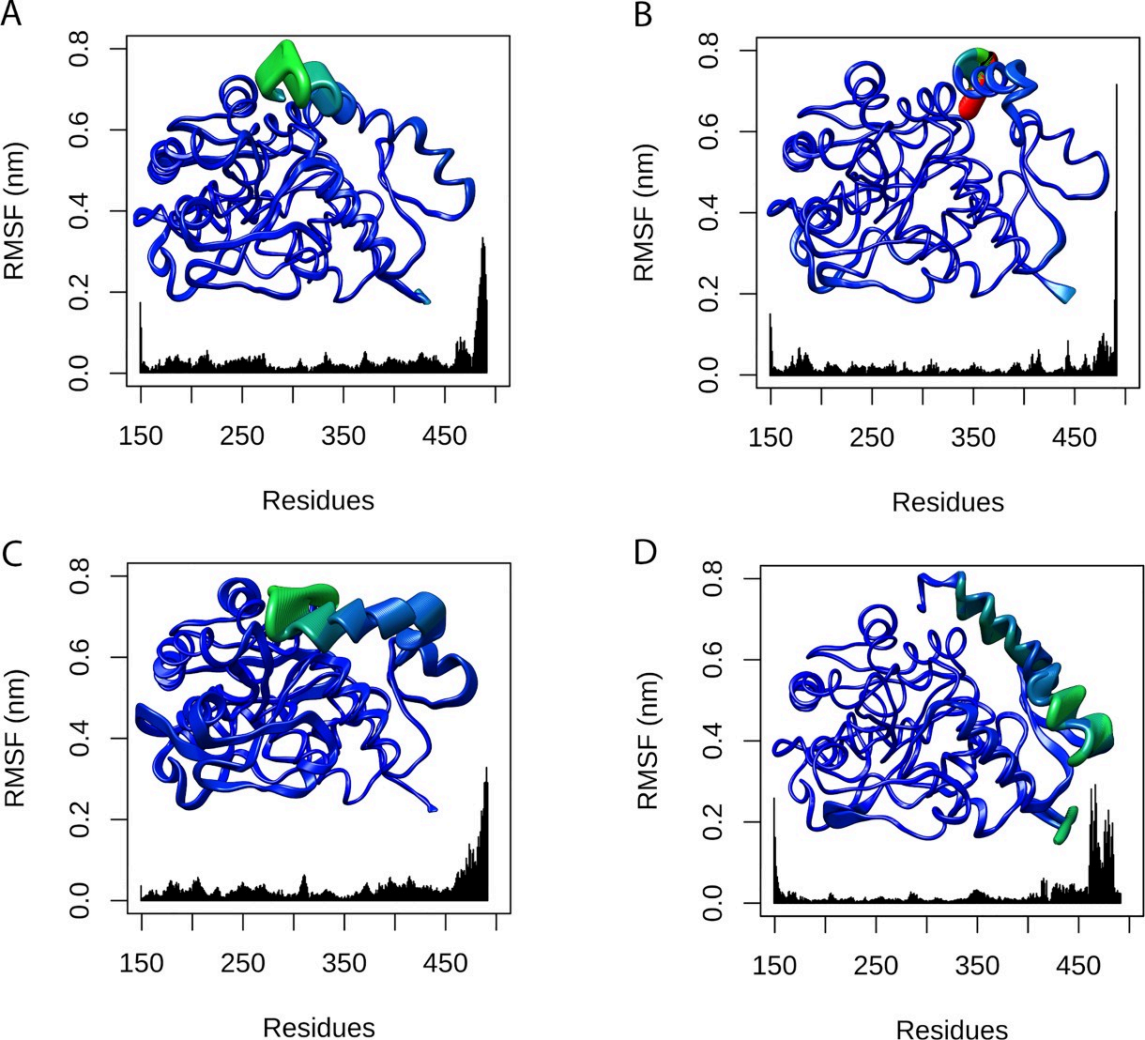
RMSF contribution to PC1 of WT TPH2 and its variants. The RMSF contribution of each protein residue to PC1 is shown as a line plot and projected on the corresponding structure. Each residue of TPH2 WT and variants was colored and sized according to its RMSF contribution. The coloring-thickness scale varies from dark blue and thin (low fluctuations) to red and thick (high fluctuations). (A) RMSF contribution of WT TPH2 to PC1. (B) RMSF contribution of variant P206S to PC1. (C) RMSF contribution of variant R303W to PC1. (D) RMSF contribution of variant R441H to PC1.

**Fig 9:**
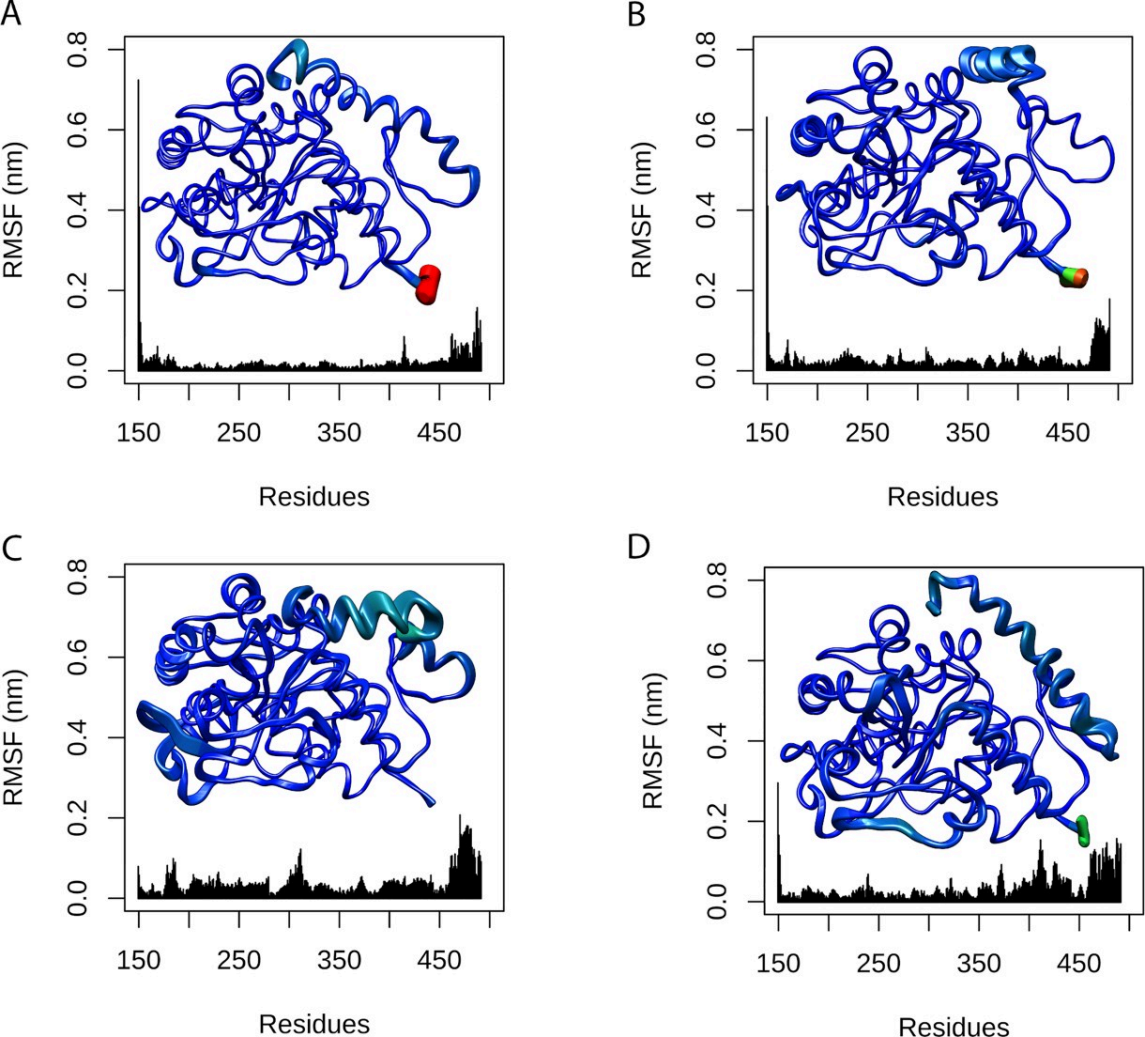
RMSF contribution to PC2 of WT TPH2 and its variants. The RMSF contribution of each protein residue to PC2 is shown as a line plot and projected on the corresponding structure. Each residue of TPH2 WT and variants was colored and sized according to its RMSF contribution. The coloring-thickness scale varies from dark blue and thin (low fluctuations) to red and thick (high fluctuations). (A) RMSF contribution of WT TPH2 to PC2. (B) RMSF contribution of variant P206S to PC2. (C) RMSF contribution of variant R303W to PC2. (D) RMSF contribution of variant R441H to PC2.

We then performed PCA of TPH2 WT and its variants. As shown in S1 Fig, the first two PCs (PC1 and PC2) captured the dominant motions, accounting for 44.49%, 32.29%, 58.52%, and 53.66% of the total variance for WT TPH2 and its variants P206S, R303W, and R441H, respectively. We analyzed the projections of the MD trajectories onto the subspace spanned by PC1 and PC2 (Fig 7), which suggested alterations in the essential dynamics of all variants when compared to the WT. We also analyzed the RMSF contribution of each protein residue to PC1 (Fig 8) and PC2 (Fig 9), which pointed to essential mobility alterations at the catalytic and oligomerization domains of R303W and R441H. Essential mobility alterations were also observed at the oligomerization domain of P206S. The PCA analysis thus suggested that the analyzed variants could affect the essential dynamics of TPH2.

Rg is a measure of the structural displacement of protein atoms from their common center of mass, thereby providing information on protein compactness throughout the simulation. Rg analysis is then useful to describe the overall dimensions of proteins [25]. The Rg values computed during the simulations for WT TPH2 and its variants are shown in S2 Fig. A sudden increase followed by a rapid decrease in the Rg values was observed in the first nanoseconds of all simulations. After this initial moment of structural instability, the Rg values assumed a steady behavior until the end of the trajectories, except for the R441H variant. The Rg values for WT TPH2 (2.006±0.007 nm) are higher than those of P206S (1.990±0.006 nm); and similar to those of R303W (2.021±0.009 nm) and R441H (1.993±0.011 nm). This result suggests compactness alterations in the P206S variant.

SASA is a measure of the exposed surface of a given protein [29], thereby providing information on the protein’s ability to interact with the solvent [15]. The SASA values computed throughout the simulations are shown in S3 Fig. The SASA values of the analyzed structures presented an unstable behavior at the beginning of the simulation. After approximately 30 ns, the SASA values assumed a steady behavior until the end of the MD trajectories, except for the R441H variant. The SASA values for WT TPH2 (169.70±2.64 nm^2^) are similar to those of P206S (165.12±219 nm^2^), R303W (171.95±2.78 nm^2^), and R441H (165.52±3.89 nm^2^). This result indicates that the analyzed variants may not affect the TPH2 exposed surface.

**Fig 10:**
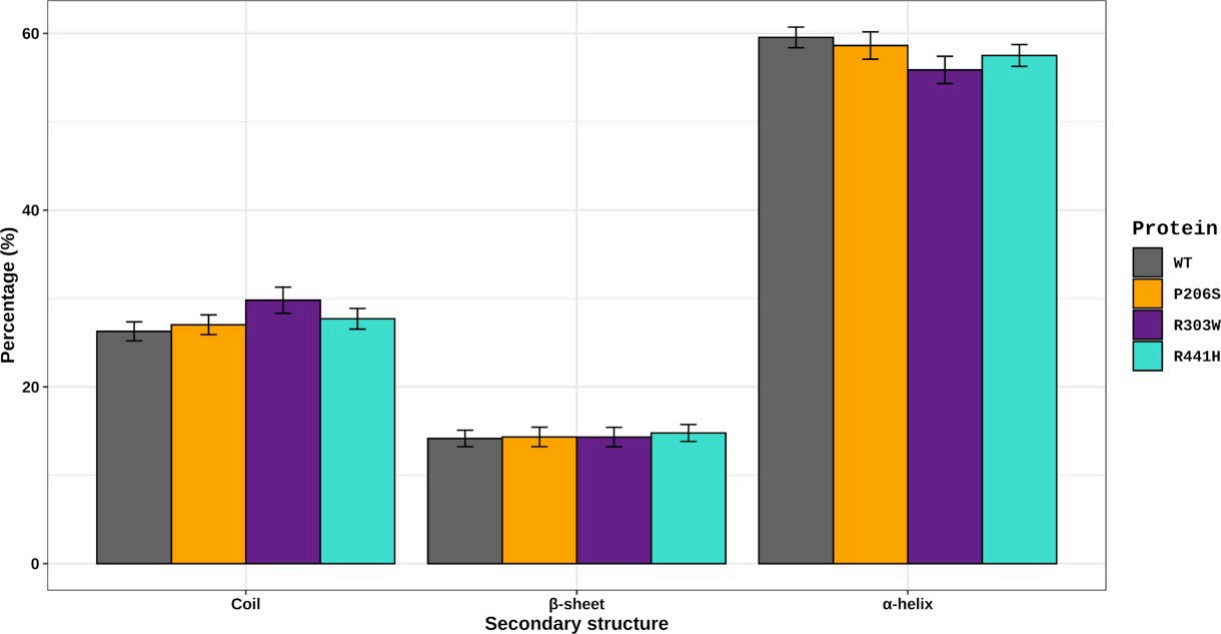
Secondary structure analysis of TPH2 WT and its variants. The average number (in percentages) of alpha-helices, beta-sheets and coils formed throughout the simulations of WT TPH2 and its variants P206S, R303W, and R441H are shown as a bar plot. The error bar represents the standard deviation. The bar plot was designed using the *ggplot2* library implemented in R software.

SS analysis was also performed to further understand the structural impact of P206S, R303W, and R441H mutations in the TPH2 protein. The average number (in percentage) of alpha-helices, beta-sheets, and coils formed at the equilibrium state in all simulations are shown in Fig 10. The number of alpha-helices formed during the WT simulation (59.5±1.1%) is higher than that of R303W (55.8±1.5%) and similar to those of P206S (58.6±1.5%) and R441H (57.5±1.2%). The number of coils formed during the WT simulation (26.3±1.1%) is lower than that of R303W (29.8±1.5) and similar to those of P206S (27.0±1.1%) and R441H (27.7±1.1%). The number of β-sheets formed during the WT (14.1±0.9%) simulation is similar to those of P206S (14.3±1.1%), R303W (14.3±1.1%), and R441H (14.3±1.1%). This analysis thus suggests secondary structure alterations at the R303W variant.

As previously shown (Figs 5–9), flexibility and essential mobility alterations were observed at the oligomerization domain of the studied TPH2 variants. We then analyzed the number of hydrogen bonds and hydrophobic contacts formed between the TPH2 monomers throughout the simulations to further investigate the impact of P206S, R303W, and R441H amino acid substitutions on dimer coordination. We also performed MMPBSA analysis of TPH2 WT and its variants for this purpose.

**Fig 11:**
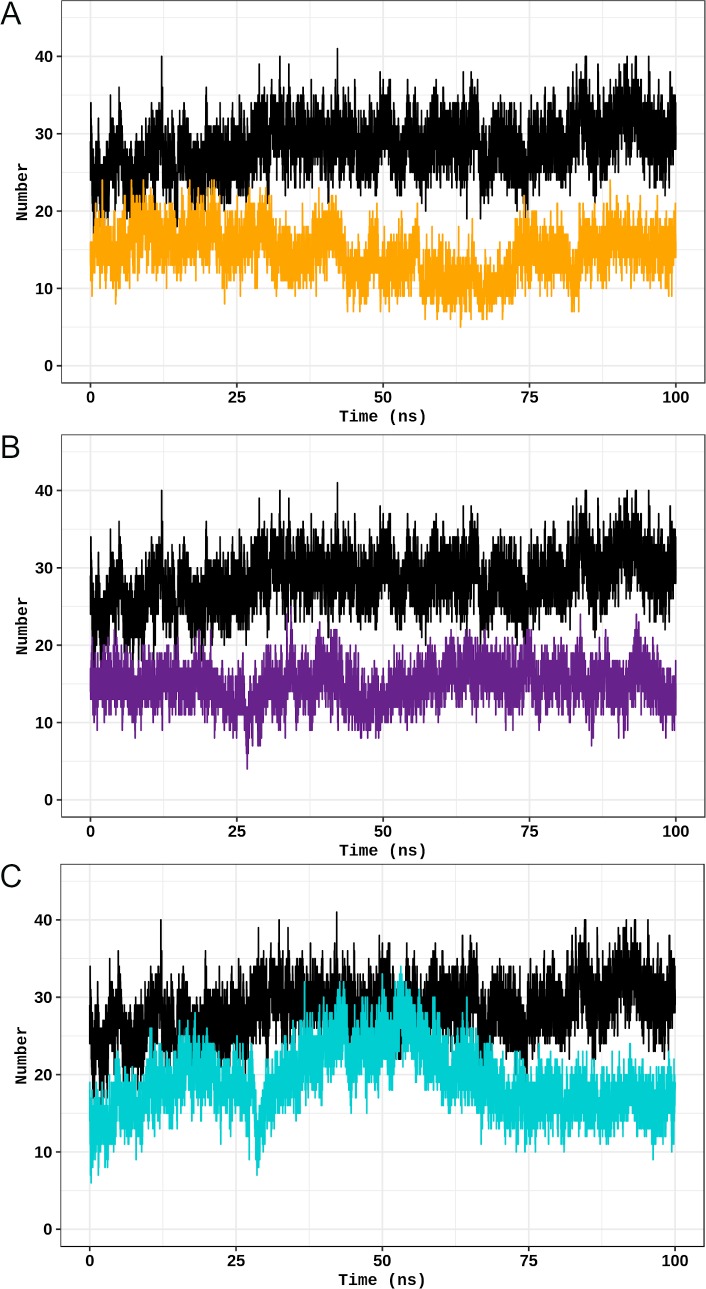
Hydrogen bond analysis of TPH2 WT and its variants. The number of hydrogen bonds formed during the simulations of TPH2 WT and its variants is shown as a function of time. (A) Comparison between the WT (black) and P206S variant (dark yellow). (B) Comparison between the WT (black) and R303W variants (purple). (C) Comparison between the WT (black) and R441H variant (turquoise).

**Fig 12:**
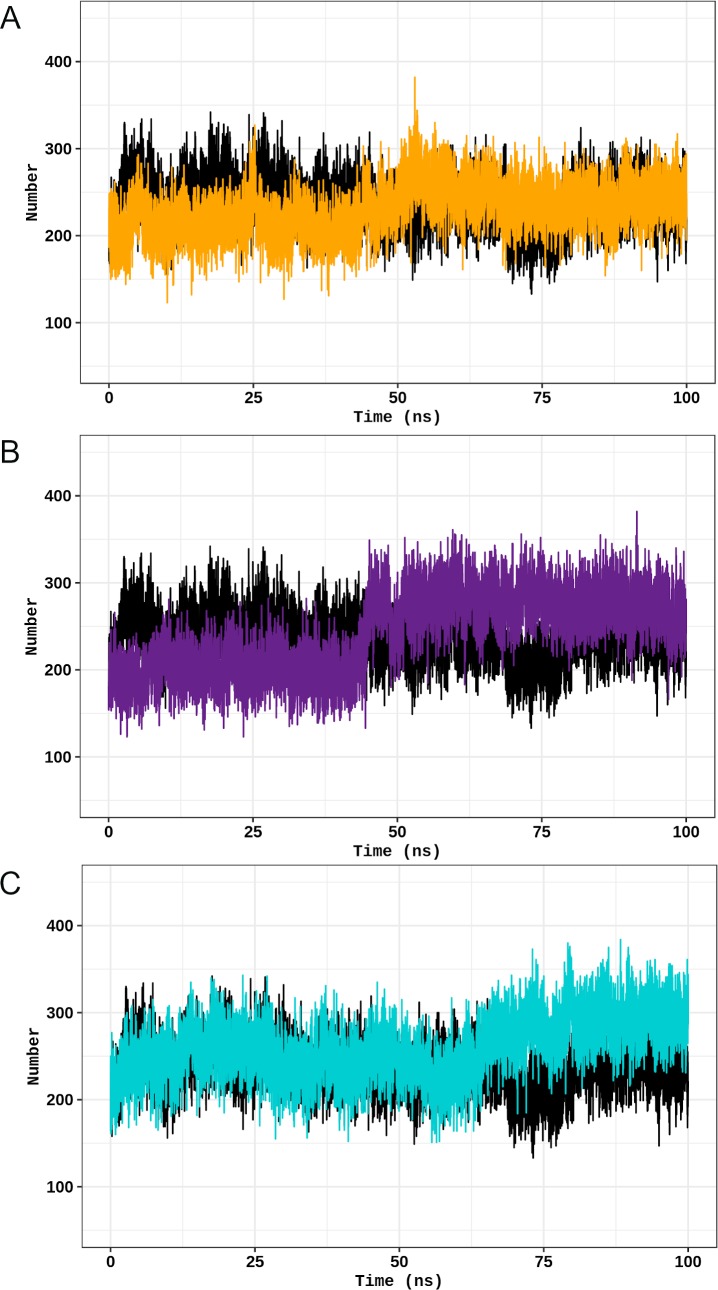
Hydrophobic contact analysis of TPH2 WT and its variants. The number of hydrophobic contacts formed during the simulations of TPH2 WT and its variants is shown as a function of time. (A) Comparison between the WT (black) and P206S variant (dark yellow). (B) Comparison between the WT (black) and R303W variants (purple). (C) Comparison between the WT (black) and R441H variant (turquoise).

The stability of a protein is determined by its number of interactions [15], particularly hydrogen bonds and hydrophobic contacts [30]. Thus, the numbers of interactions carried out between the TPH2 monomers are proportional to dimer stability. The number of hydrogen bonds and hydrophobic contacts formed during the simulations of TPH2 WT and its variants over time is shown in Fig 11 and Fig 12, respectively. The number of hydrogen bonds formed in all simulations presented a steady behavior after approximately 30 ns (Fig 11), except for the R441H variant. The number of hydrogen bonds formed during the WT (29.37±3.06) simulation is higher than those of P206S (13.77±2.84), R303W (15.21±2.48), and R441H (19.81±4.11). The number of hydrophobic contacts formed between TPH2 WT and its variants, which is shown in Fig 12, also presented a steady behavior. The number of hydrogen bonds formed between the wild-type TPH2 monomers (233.14±26.77) is similar to those of P206S (236.95±28.33), R303W (259.82±40.67), and R441H (264.13±36.84) variants. Thus, the hydrogen bond analysis may have indicated stability alterations in the studied TPH2 variants.

The strength of a biomolecular interaction can be analyzed by quantifying its binding free energy. Actually, a variety of computational approaches can be used to estimate binding free energies, including the MM-PBSA method (**m**olecular **m**echanics **P**oisson−**B**oltzmann **s**urface **a**rea), which is widely used to compute interaction energies and is often applied to the study of biomolecular complexes [24]. The MM-PBSA method also allows addressing contributions of electrostatic and vdW interactions to binding affinity [31]. According to the MM-PBSA analysis, whose results are shown in Table 1, the average total binding free energy of the WT TPH2 dimer is similar to that of R303W and higher than that of the P206S and R441H variants. Thus, the P206S and R441H variants may affect the affinity of TPH2 dimer interactions.

**Table 1:** MM-PBSA analysis of WT TPH2 and its variants.

Energy term (kJ/mol)	WT	P206S	R303W	R441H
**Van der Waals**	-928.23 (0.47)	-888.58 (0.59)	-1023.65 (0.82)	-938.13 (0.52)
**Electrostatic**	-1609.73 (3.18)	-1667.54 (3.94)	-1117.19 (2.93)	-2044.99 (3.74)
**Total**	-2537.97 (3.22)	-2556.12 (4.08)	-2140.85 (2.98)	-2983.12 (3.70)

The standard errors of the mean are given in parentheses.

## Discussion

The TPH2 enzyme catalyzes the first step and rate-limiting reaction of serotonin biosynthesis [6,7]. Serotonin is indispensable for the regulation of several homeostatic processes related to sleep, mood, and food intake [9]. Thus, alterations in serotonin levels and central serotonergic system deregulation are known to cause brain dysfunction, leading to the development of PD [10]. As the rate-limiting step of this neurotransmitter biosynthesis, mutations in TPH2 have been associated with several PD, such as bipolar disorder, depression, and attention deficit-hyperactivity disorder [11].

Although millions of novel genetic variants are currently being discovered by next-generation sequencing projects [18], the characterization of their effects is extremely expensive, time-consuming and difficult when using wet-lab experiments [16]. Computational prediction methods, which are less expensive and faster but accurate, allow the prioritization of the most likely disease-related mutations to be thoroughly examined with experiments, thereby saving time and resources [14]. Computational prediction methods can also be applied to the study of already known disease-related mutations. This approach provides insight into the molecular mechanisms of pathology and furnishes relevant information that could lead to improvements in existing treatments [17]. Computational methods have thus become an essential approach for the study of genetic variants [15].

The effects of deleterious mutations on proteins can be predicted using computational methods [25]. I-Mutant disease, Mutpred, PhD-SNP, nsSNP-Analyzer, Pmut, SNP&GO, SNAP2, Polyphen-2, SNPEffect4.0 [15], and VarMod [21] were used to predict the functional and structural effects of TPH2 mutations.

The functional prediction analysis showed that the variants P206S, R303W, and R441H, which had been previously associated with PD [11], were predicted as deleterious by 44%, 100% and 89% of the algorithms, respectively (Fig 2). This analysis pointed to the low accuracy of the algorithms used in detecting the known deleterious effects of the P206S mutation. These algorithms were trained in different datasets and analyzed different parameters to make predictions. Moreover, there is no established gold standard method for predicting the functional effects of mutations. Thus, as previously shown by our group, using a variety of algorithms is indispensable in predicting the functional effects of mutations [15,20].

The functional prediction analysis (Fig 2) also showed that the variants G345E, E363K, and S383F were predicted as deleterious by 100% of the algorithms used, thereby suggesting that they could be harmful to TPH2. On the other hand, the variants V78I, L83V, M91I, Q124R, and E145Q were predicted as neutral for 100% of the algorithms used, which suggests that their effects may be neutral for TPH2 function. This analysis also showed that the TPH2 mutations predicted as deleterious by most of the algorithms used mainly affect its catalytic domain rather than the regulatory domain (Fig 2), which reinforces the functional importance of the residues within the TPH2 catalytic domain.

The SNPeffect prediction (S2 Table) indicated that the variants T64I, R225Q, S383F, and R441H increase protein aggregation, while the variants R82S, and M91I increase amyloid propensity, and the variant R225Q increases chaperone binding.

The stability prediction analysis (S3 Table) indicated that the variants P206S, R276S, P277L, R430G, M432I, A436E, R441H, R471H, and L474V reduce TPH2 stability, while the variants R383F, D479E, and Q486K do not affect its stability. The other TPH2 variants, including R303W, presented discordant results in the stability prediction (S3 Table), which may occur because these algorithms apply different methods to make predictions. FoldX estimates the free energy variation upon mutation by applying an empirical force field trained in a database of molecular interactions of engineered proteins, while I-Mutant uses a Support Vector Machine trained in a dataset of experimentally determined structures to estimate the free energy variation upon mutation [15].

Relevant residues for protein structure and function are usually conserved throughout evolution due to the high selective pressure [32]. The biological importance of an amino acid residue can therefore be related to its evolutionary conservation level [22]. We then calculated the evolutionary conservation of each residue of TPH2 using the ConSurf Server (Fig 3). According to ConSurf, the positions R303 and R441 were classified as highly conserved (Fig 3A), thereby suggesting functional importance. The variants R303W and R441H decrease protein stability, solubility and catalytic activity, ultimately leading to protein dysfunction. These variants were also associated with the development of PD. The position P206, in turn, was classified as variable (Fig 3A). However, the variant P206S affects protein stability and solubility, resulting in loss of function. This mutation has also been associated with the development of PD [11].

ConSurf also classified the positions affected by the mutations S22L, E363K, A378T, and S383F as highly conserved (Fig 3B). This classification suggests that these variants may be deleterious to TPH2, since they affect possibly important residues to protein, particularly the R363K mutation, which was also predicted as deleterious by 100% of the functional prediction algorithms used (Fig 2). Moreover, the ConSurf analysis indicated that the mutations affecting the TPH2 catalytic domain usually occur at conserved positions (Fig 3B), and these mutations were also predicted as deleterious by most of the functional prediction algorithms used (Fig 2), thereby suggesting functional importance.

Furthermore, the low accuracy of the functional prediction algorithms used in detecting the known deleterious potential of P206S mutation (Fig 2) could be related to the variable conservation of position P206 in TPH2 (Fig 3A), since most of the functional prediction algorithms use evolutionary information from the sequence to make predictions [20].

The structural impact of mutations affecting specific amino acids can be narrowly examined using MD simulations. This approach is useful and widely used in the computational study of biomolecules, since it provides detailed information on changes in protein conformation over time that can be used to analyze several structural parameters, including hydrogen bonding, flexibility, compactness and accessible surface [15]. The characterization of structural parameters in protein variants constitutes an important approach used to understand the impact of missense mutations [25], which favors understanding their corresponding mechanism of disease and may contribute to identifying more efficient treatments [33].

We then performed MD simulations of the TPH2 WT and its variants P206S, R303W and R441H using the GROMACS 5.0.7 package. RMSD analysis (Fig 4) indicated that TPH2 WT and its variants P206S, R303W and R441H float to a stable conformation, thereby suggesting system equilibration [25]. High fluctuations in RMSD (Fig 4), Rg (S2 Fig), and SASA (S3 Fig) values were observed in the first 30 ns of the WT, P206S and R303W simulations. System equilibration usually occurs after an initial moment of structural instability, in which the protein is still adapting to the force field used in the simulations [15].

The RMSF (Fig 5) and B-factor (Fig 6) analyses pointed to flexibility alterations at the oligomerization domain of all analyzed TPH2 variants, in addition to flexibility increase at the catalytic domain of R303W and R441H variants. The flexibility alterations were particularly high at the oligomerization domain of R303W and R441H. The RMSF and B-factor analyses, thus, suggested that the analyzed variants, especially R303W and R441H, could affect TPH2 flexibility. Protein flexibility is determinant for binding affinity and specificity. Thus, flexibility alterations can lead to strong and nonintuitive consequences for protein binding properties [34]. The flexibility alterations observed during the MD simulations of the P206S, R303W, and R441H variants indicate that these mutations may affect TPH2 binding affinity and specificity, particularly at the oligomerization and catalytic domains.

We also performed PCA to study the influence of P206S, R303W, and R441H mutations on TPH2 molecular motions. The PCA allows the separation of protein motions into two subspaces: 1) the essential subspace, which usually describes relevant movements to protein function, such as opening, closing, and flexing; and 2) the remaining subspace, which usually describes small irrelevant local fluctuations [35]. The PCA suggested that all analyzed variants could affect the TPH2 essential dynamics (Fig 7). The PCA also pointed to essential mobility alterations at the oligomerization domain of all analyzed variants in addition to essential mobility alterations at the catalytic domain of R303W and R441H (Fig 8 and Fig 9). The mobility alterations observed in the PCA occur at regions similar to those found altered in the RMSF (Fig 5) and B-factor (Fig 6) analyses.

The biological functions of proteins are usually determined by their essential motions, particularly those related to protein interactions and binding to substrates [25]. Thus, the essential mobility alterations observed in all variants (Figs 7–9) suggest that these mutations may affect TPH2 functional interactions. Moreover, the alterations observed during PCA could be related to the functional impairment of TPH2 upon P206S, R303W, and R441H mutations, since these alterations affect the oligomerization and catalytic domain, which are crucial to protein function [6]. The oligomerization process is required for TPH2 activity [36]. Thus, the flexibility (Fig 5 and Fig 6) and essential mobility alterations (Fig 7, Fig 8 and Fig 9) observed in the MD analyses, which mainly affect the oligomerization domain, may impair TPH2 oligomer interactions and formation, consequently reducing protein activity. The mutations P206S, R303W, and R441H have already been reported to reduce TPH2 activity [11]. However, an *in vitro* study using PC12 cells, a cell line derived from a pheochromocytoma of the rat adrenal medulla [37], showed that the mutation R441H does not impair TPH2 oligomerization [36]. Thus, to further investigate the impact of P206S, R303W, and R441H amino acid substitutions on dimer coordination, we performed MM-PBSA analysis of TPH2 WT and its variants, as well as analyzed the number of hydrogen bonds and hydrophobic contacts formed between the TPH2 monomers throughout the simulations. The MM-PBSA analysis can estimate the binding free energy involved in dimer interaction and, consequently, its binding affinity [24]. In addition, the number of interactions, such as hydrogen bonds and hydrophobic contacts, is a crucial factor in protein stability [15]. These MD analyses could therefore provide relevant information on TPH2 dimer coordination.

The hydrogen bond analysis (Fig 11) indicated that the P206S, R303W, and R441H variants formed fewer interdimeric hydrogen bonds throughout the simulation than the WT. Thus, these variants may change TPH2 dimer stability [38]. No alterations were observed in the number of interdimeric hydrophobic contacts formed over the WT and its variant simulations (Fig 12). The MM-PBSA analysis (Table 1) pointed to alterations in the total binding free energy of P206S and R441H dimers when compared to the WT, which indicated that these variants may affect the binding affinity of TPH2 dimers [24]. The MM-PBSA also showed that the electrostatic term is the main contributing factor to the total binding free energy alterations observed during the P206S and R441H simulations. Changes in protein-protein binding affinity and stability caused by mutations, including those affecting dimer interactions, pose a high risk of being deleterious [39]. Thus, the hydrogen bond and MM-PBSA analyses suggested that the analyzed TPH2 variants may affect dimer coordination with probably deleterious consequences to the protein.

The Rg (S2 Fig) and SASA (S3 Fig) analyses pointed to compactness alterations in the P206S variant but no alterations in the accessible surface area. This result thus suggests that the P206S mutation could affect the surface-to-volume ratio of TPH2, which is also associated with the protein’s ability to interact [15]. Furthermore, steady RG values during the simulation suggest a stable protein folding [29], which was observed after 30 ns for WT TPH2 and its P206S and R303W variants (S2 Fig).

The SS analysis pointed to alterations in the average number of alpha-helices and coils formed during the R303W variant simulation. SS alterations have also been reported in the P206S variant of TPH2 [8]. In addition to protein flexibility, SS is also an important factor for protein interactions. Alterations in this structural pattern may affect protein recognition and interactions, which could impact its function [25]. The alterations in the SS of R303W further suggest that this mutation may affect TPH2 recognition and interactions. Moreover, TPH2 has three catalytic iron-binding sites at positions 318, 323 and 363 [11]. The amino acid substitution R303W occurs at 14.4 Å [8] from the closest iron-binding site. The RMSF (Fig 5), B-factor (Fig 6), and PC analyses (Fig 7, Fig 8 and Fig 9) showed that this mutation affects the residue 303 and its surroundings, thereby increasing their flexibility and essential mobility when compared to WT TPH2. This mutation could then impair the enzyme activity by affecting the interactions of residue 303 and its surroundings, resulting in TPH2 activity loss and functional impairment, which have already been reported in the literature [11].

The MD analyses, thus indicated flexibility and essential mobility alterations especially at the oligomerization domain of the analyzed variants. The variants R303W and R441H also presented flexibility and essential mobility alterations at the catalytic domain. Furthermore, secondary structure alterations were observed in the R303W variant. Secondary structure formation, protein flexibility, and essential dynamics are determinants of protein interactions [25,34]. The analyzed variants either presented alterations in dimer binding affinity and stability throughout the simulations. Thus, the alterations observed in the MD analyses suggest that these variants may affect TPH2 interactions particularly at the oligomerization and catalytic domains, which are crucial to protein function [6]. This finding may therefore be related to the functional impairment of TPH2 upon P206S, R303W, and R441H mutations, as well as their involvement in psychiatric disorders [11].

Furthermore, we developed a database, SNPMOL (http://www.snpmol.org/) [40], to host the results presented in this paper. The database is human-curated and freely available for biologists and clinicians to exploit the functional and structural effects of the 46 TPH2 variants that we compiled from the literature.

## Conclusions

Forty-six TPH2 variants were compiled from the literature and analyzed by functional and stability prediction algorithms. Among the analyzed variants, those occurring at the catalytic domain were noticeably more damaging to protein structure and function, including the variants P206S, R303W and R441H, which had already been reported to be associated with psychiatric disorders. The ConSurf analysis indicated that the mutations affecting the catalytic domain were also more conserved throughout evolution. Additionally, the variants S364K and S383F were predicted to be deleterious by all the functional algorithms used and occur at highly conserved positions, which indicates that they might be deleterious and, consequently, valuable targets for future investigation. The MD analyses indicate that the variants P206S, R303W, and R441H affect TPH2 flexibility, essential mobility, and secondary structure, particularly at the catalytic and oligomerization domains, which are crucial to protein function. The variants P206S, R303W, and R441H also presented alterations in dimer binding affinity and stability throughout the MD simulations that we performed. Thus, considering that these parameters are determinant for protein interactions, the alterations observed throughout the MD simulations may be related to the functional impairment of TPH2 upon P206S, R303W, and R441H mutations, as well as their involvement in psychiatric disorders. Understanding the effects of TPH2 mutations on protein structure and function may lead to improvements in existing treatments for psychiatric disorders and facilitate the design of further experiments.

## Supporting information

S1 Table Functional prediction of each TPH2 protein variant. (DOCX)

S2 Table Prediction of amyloid propensity, chaperone binding, and protein aggregation tendency for each TPH2 protein variant. (DOCX)

S3 Table Stability prediction of each TPH2 protein variant. (DOCX)

S1 Fig Percentage of the total variance explained by PC1 and PC2 for TPH2 WT and its variants. Projection of PC1 and PC2 extracted from the essential dynamics and the percentage of the total variance explained by them. The trajectory frames for TPH2 WT and variants are colored from blue to red according to time evolution. (A) PCA plot of WT TPH2. The first two PCs account for 44.49% of the total variance. (B) PCA plot of variant P206S. The first two PCs account for 32.29% of the total variance. (C) PCA plot of variant R303W. The first two PCs account for 58.52% of the total variance. (D) PCA plot of variant R441H. The first two PCs account for 53.66% of the total variance.
(TIF)

S2 Fig Rg of WT TPH2 and its variants. The Rg values of WT TPH2 and its variant at 300 K are shown as a function of time. (A) Comparison between the WT (black) and P206S variant (dark yellow). (B) Comparison between the WT (black) and R303W variants (purple). (C) Comparison between the WT (black) and R441H variant (turquoise).
(TIF)

S3 Fig SASA of WT TPH2 and its variants. The SASA values of WT TPH2 and its variant at 300 K are shown as a function of time. (A) Comparison between the WT (black) and P206S variant (dark yellow). (B) Comparison between the WT (black) and R303W variant (purple). (C) Comparison between the WT (black) and R441H variant (turquoise).
(TIF)
