# Supplementary material for: In silico analysis of the tryptophan hydroxylase 2 (TPH2) protein variants related to psychiatric disorders
Source: PLoS One. 2020 Mar 2;15(3):e0229730. doi: 10.1371/journal.pone.0229730 (PMC7051086; doi:10.1371/journal.pone.0229730)
Supplement: S1 Table — (DOCX) [file pone.0229730.s001.docx]

**S1 Table. Functional prediction of each TPH2 protein variant.**

| **Protein Variant / dbSNP ID** | **PhD-SNP** | **SNP&GO** | **I-Mutant Disease** | **nsSNP-Analyzer** | **Pmut** | **MutPred** | **SNAP2** | **Polyphen2** | **VarMod** |
| --- | --- | --- | --- | --- | --- | --- | --- | --- | --- |
| [S22L/rs374275719](http://www.ncbi.nlm.nih.gov/projects/SNP/snp_ref.cgi?rs=374275719) | Deleterious | Deleterious | Deleterious | Deleterious | Neutral | Neutral | Neutral | Deleterious | Neutral |
| [P25L/rs150922091](http://www.ncbi.nlm.nih.gov/projects/SNP/snp_ref.cgi?rs=150922091) | Neutral | Neutral | Deleterious | Neutral | Neutral | Neutral | Neutral | Neutral | Neutral |
| [G32D/rs201958204](http://www.ncbi.nlm.nih.gov/projects/SNP/snp_ref.cgi?rs=201958204) | Deleterious | Deleterious | Deleterious | Neutral | Deleterious | Neutral | Neutral | Neutral | Neutral |
| [L36V/rs34115267](http://www.ncbi.nlm.nih.gov/projects/SNP/snp_ref.cgi?rs=34115267) | Neutral | Neutral | Neutral | Neutral | Neutral | Neutral | Deleterious | Neutral | Neutral |
| [L36P/rs199775778](http://www.ncbi.nlm.nih.gov/projects/SNP/snp_ref.cgi?rs=199775778) | Deleterious | Neutral | Deleterious | Neutral | Deleterious | Neutral | Neutral | Neutral | Neutral |
| [S41Y/rs78162420](http://www.ncbi.nlm.nih.gov/projects/SNP/snp_ref.cgi?rs=78162420) | Neutral | Neutral | Deleterious | Neutral | Neutral | Neutral | Deleterious | Neutral | Neutral |
| [S53R/rs146693226](http://www.ncbi.nlm.nih.gov/projects/SNP/snp_ref.cgi?rs=146693226) | Neutral | Neutral | Neutral | Neutral | Deleterious | Neutral | Neutral | Neutral | Neutral |
| [R55C/rs75558144](http://www.ncbi.nlm.nih.gov/projects/SNP/snp_ref.cgi?rs=75558144) | Neutral | Neutral | Deleterious | Deleterious | Neutral | Neutral | Deleterious | Neutral | Neutral |
| [T64I/rs373761659](http://www.ncbi.nlm.nih.gov/projects/SNP/snp_ref.cgi?rs=373761659) | Neutral | Neutral | Neutral | Neutral | Neutral | Deleterious | Neutral | Neutral | Neutral |
| [A65T/rs557063960](http://www.ncbi.nlm.nih.gov/projects/SNP/snp_ref.cgi?rs=557063960) | Neutral | Neutral | Deleterious | Neutral | Neutral | Neutral | Neutral | Neutral | Neutral |
| [V78I/rs200000346](http://www.ncbi.nlm.nih.gov/projects/SNP/snp_ref.cgi?rs=200000346) | Neutral | Neutral | Neutral | Neutral | Neutral | Neutral | Neutral | Neutral | Neutral |
| [R82S/rs147125940](http://www.ncbi.nlm.nih.gov/projects/SNP/snp_ref.cgi?rs=147125940) | Neutral | Neutral | Deleterious | Neutral | Deleterious | Deleterious | Neutral | Neutral | Neutral |
| [L83V/rs148074013](http://www.ncbi.nlm.nih.gov/projects/SNP/snp_ref.cgi?rs=148074013) | Neutral | Neutral | Neutral | Neutral | Neutral | Neutral | Neutral | Neutral | Neutral |
| [E86Q/rs77828767](http://www.ncbi.nlm.nih.gov/projects/SNP/snp_ref.cgi?rs=77828767) | Neutral | Deleterious | Deleterious | Neutral | Neutral | Neutral | Neutral | Neutral | Neutral |
| [R88H/rs138035638](http://www.ncbi.nlm.nih.gov/projects/SNP/snp_ref.cgi?rs=138035638) | Neutral | Neutral | Deleterious | Neutral | Neutral | Neutral | Neutral | Neutral | Neutral |
| [M91I/rs146967917](http://www.ncbi.nlm.nih.gov/projects/SNP/snp_ref.cgi?rs=146967917) | Neutral | Neutral | Neutral | Neutral | Neutral | Neutral | Neutral | Neutral | Neutral |
| [R100Q/rs530018658](http://www.ncbi.nlm.nih.gov/projects/SNP/snp_ref.cgi?rs=530018658) | Neutral | Neutral | Deleterious | Neutral | Deleterious | Neutral | Neutral | Neutral | Neutral |
| [R101Q/rs149529607](http://www.ncbi.nlm.nih.gov/projects/SNP/snp_ref.cgi?rs=149529607) | Neutral | Neutral | Deleterious | Neutral | Deleterious | Neutral | Neutral | Neutral | Neutral |
| [Q124R/rs548166835](http://www.ncbi.nlm.nih.gov/projects/SNP/snp_ref.cgi?rs=548166835) | Neutral | Neutral | Neutral | Neutral | Neutral | Neutral | Neutral | Neutral | Neutral |
| [T134M/rs560203761](http://www.ncbi.nlm.nih.gov/projects/SNP/snp_ref.cgi?rs=560203761) | Neutral | Deleterious | Neutral | Deleterious | Neutral | Neutral | Deleterious | Neutral | Neutral |
| [E145Q/rs527350890](http://www.ncbi.nlm.nih.gov/projects/SNP/snp_ref.cgi?rs=527350890) | Neutral | Neutral | Neutral | Neutral | Neutral | Neutral | Neutral | Neutral | Neutral |
| [R156Q/rs147469527](http://www.ncbi.nlm.nih.gov/projects/SNP/snp_ref.cgi?rs=147469527) | Deleterious | Deleterious | Deleterious | Neutral | Deleterious | Deleterious | Deleterious | Neutral | Neutral |
| [P206S/rs17110563](http://www.ncbi.nlm.nih.gov/projects/SNP/snp_ref.cgi?rs=17110563) | Deleterious | Deleterious | Deleterious | Neutral | Neutral | Neutral | Deleterious | Neutral | Neutral |
| [R225Q/rs139896303](http://www.ncbi.nlm.nih.gov/projects/SNP/snp_ref.cgi?rs=139896303) | Neutral | Deleterious | Deleterious | Neutral | Deleterious | Deleterious | Neutral | Neutral | Neutral |
| [R276S/rs773797105](https://www.ncbi.nlm.nih.gov/snp/rs773797105) | Deleterious | Deleterious | Deleterious | Deleterious | Neutral | Deleterious | Deleterious | Deleterious | Neutral |
| [P277L/rs373088979](http://www.ncbi.nlm.nih.gov/projects/SNP/snp_ref.cgi?rs=373088979) | Deleterious | Deleterious | Deleterious | Deleterious | Neutral | Deleterious | Deleterious | Deleterious | Deleterious |
| [R303W/rs120074176](http://www.ncbi.nlm.nih.gov/projects/SNP/snp_ref.cgi?rs=120074176) | Deleterious | Deleterious | Deleterious | Deleterious | Deleterious | Deleterious | Deleterious | Deleterious | Deleterious |
| [A328V/rs2887147](http://www.ncbi.nlm.nih.gov/projects/SNP/snp_ref.cgi?rs=2887147) | Neutral | Deleterious | Neutral | Neutral | Deleterious | Neutral | Neutral | Deleterious | Neutral |
| [I339M/rs143153059](https://www.ncbi.nlm.nih.gov/snp/rs143153059) | Neutral | Neutral | Neutral | Neutral | Neutral | Deleterious | Neutral | Deleterious | Neutral |
| [G345E/rs142857022](http://www.ncbi.nlm.nih.gov/projects/SNP/snp_ref.cgi?rs=142857022) | Deleterious | Deleterious | Deleterious | Deleterious | Deleterious | Deleterious | Deleterious | Deleterious | Deleterious |
| [D348H/rs370276646](http://www.ncbi.nlm.nih.gov/projects/SNP/snp_ref.cgi?rs=370276646) | Deleterious | Deleterious | Deleterious | Deleterious | Deleterious | Deleterious | Neutral | Deleterious | Deleterious |
| [E363K/rs146667593](http://www.ncbi.nlm.nih.gov/projects/SNP/snp_ref.cgi?rs=146667593) | Deleterious | Deleterious | Deleterious | Deleterious | Deleterious | Deleterious | Deleterious | Deleterious | Deleterious |
| [A378T/rs78369038](http://www.ncbi.nlm.nih.gov/projects/SNP/snp_ref.cgi?rs=78369038) | Deleterious | Deleterious | Neutral | Neutral | Neutral | Deleterious | Deleterious | Deleterious | Deleterious |
| [S383F/rs371065822](http://www.ncbi.nlm.nih.gov/projects/SNP/snp_ref.cgi?rs=371065822) | Deleterious | Deleterious | Deleterious | Deleterious | Deleterious | Deleterious | Deleterious | Deleterious | Deleterious |
| [C396R/rs533935200](http://www.ncbi.nlm.nih.gov/projects/SNP/snp_ref.cgi?rs=533935200) | Neutral | Deleterious | Deleterious | Neutral | Neutral | Neutral | Neutral | Neutral | Neutral |
| [T404K/rs191507707](http://www.ncbi.nlm.nih.gov/projects/SNP/snp_ref.cgi?rs=191507707) | Neutral | Neutral | Deleterious | Neutral | Neutral | Neutral | Neutral | Neutral | Neutral |
| [E430G/rs371729573](http://www.ncbi.nlm.nih.gov/projects/SNP/snp_ref.cgi?rs=371729573) | Deleterious | Deleterious | Neutral | Neutral | Neutral | Deleterious | Deleterious | Deleterious | Deleterious |
| [M432I/rs375357832](http://www.ncbi.nlm.nih.gov/projects/SNP/snp_ref.cgi?rs=375357832) | Neutral | Neutral | Neutral | Deleterious | Neutral | Deleterious | Neutral | Neutral | Neutral |
| [A436E/rs375168296](http://www.ncbi.nlm.nih.gov/projects/SNP/snp_ref.cgi?rs=375168296) | Deleterious | Deleterious | Deleterious | Deleterious | Deleterious | Deleterious | Deleterious | Deleterious | Neutral |
| [R441H/rs120074175](http://www.ncbi.nlm.nih.gov/projects/SNP/snp_ref.cgi?rs=120074175) | Deleterious | Deleterious | Deleterious | Deleterious | Deleterious | Neutral | Deleterious | Deleterious | Deleterious |
| [R471H/rs138642957](https://www.ncbi.nlm.nih.gov/snp/rs138642957) | Neutral | Deleterious | Deleterious | Neutral | Deleterious | Deleterious | Deleterious | Deleterious | Neutral |
| [R471K/rs150148746](http://www.ncbi.nlm.nih.gov/projects/SNP/snp_ref.cgi?rs=150148746) | Neutral | Neutral | Neutral | Neutral | Neutral | Deleterious | Neutral | Neutral | Neutral |
| [D473N/rs147025898](http://www.ncbi.nlm.nih.gov/projects/SNP/snp_ref.cgi?rs=147025898) | Deleterious | Deleterious | Deleterious | Deleterious | Deleterious | Deleterious | Deleterious | Deleterious | Neutral |
| [L474V/rs199726216](http://www.ncbi.nlm.nih.gov/projects/SNP/snp_ref.cgi?rs=199726216) | Neutral | Deleterious | Deleterious | Neutral | Deleterious | Deleterious | Neutral | Deleterious | Neutral |
| [D479E/rs7488262](http://www.ncbi.nlm.nih.gov/projects/SNP/snp_ref.cgi?rs=7488262) | Neutral | Neutral | Deleterious | Neutral | Deleterious | Neutral | Neutral | Neutral | Neutral |
| [Q486K/rs545695435](http://www.ncbi.nlm.nih.gov/projects/SNP/snp_ref.cgi?rs=545695435) | Neutral | Neutral | Deleterious | Neutral | Deleterious | Neutral | Neutral | Neutral | Neutral |
