# Supplementary material for: In silico analysis of the tryptophan hydroxylase 2 (TPH2) protein variants related to psychiatric disorders
Source: PLoS One. 2020 Mar 2;15(3):e0229730. doi: 10.1371/journal.pone.0229730 (PMC7051086; doi:10.1371/journal.pone.0229730)
Supplement: S2 Table — (DOCX) [file pone.0229730.s002.docx]

**S2 Table. Prediction of amyloid propensity, chaperone binding, and protein aggregation tendency for each TPH2 protein variant.**

| **Protein Variant** | **WALTZ (amyloid propensity)** | **TANGO (aggregation propensity)** | **LIMBO (chaperone binding)** |
| --- | --- | --- | --- |
| S22L | Does not affect | Does not affect | Does not affect |
| P25L | Does not affect | Does not affect | Does not affect |
| G32D | Does not affect | Does not affect | Does not affect |
| L36V | Does not affect | Does not affect | Does not affect |
| L36P | Does not affect | Does not affect | Does not affect |
| S41Y | Does not affect | Does not affect | Does not affect |
| S53R | Does not affect | Does not affect | Does not affect |
| R55C | Does not affect | Does not affect | Does not affect |
| T64I | Increase | Does not affect | Does not affect |
| A65T | Does not affect | Does not affect | Does not affect |
| V78I | Does not affect | Does not affect | Does not affect |
| R82S | Does not affect | Increase | Does not affect |
| L83V | Does not affect | Does not affect | Does not affect |
| E86Q | Does not affect | Does not affect | Does not affect |
| R88H | Does not affect | Does not affect | Does not affect |
| M91I | Does not affect | Increase | Does not affect |
| R100Q | Does not affect | Does not affect | Does not affect |
| R101Q | Does not affect | Does not affect | Does not affect |
| Q124R | Does not affect | Does not affect | Does not affect |
| T134M | Does not affect | Does not affect | Does not affect |
| E145Q | Does not affect | Does not affect | Does not affect |
| R156Q | Does not affect | Does not affect | Does not affect |
| P206S | Does not affect | Does not affect | Does not affect |
| R225Q | Increase | Does not affect | Increase |
| R276S | Does not affect | Does not affect | Does not affect |
| P277L | Does not affect | Does not affect | Does not affect |
| R303W | Does not affect | Does not affect | Does not affect |
| A328V | Does not affect | Does not affect | Does not affect |
| I339M | Does not affect | Does not affect | Does not affect |
| G345E | Does not affect | Does not affect | Does not affect |
| D348H | Does not affect | Does not affect | Does not affect |
| E363K | Does not affect | Does not affect | Does not affect |
| A378T | Does not affect | Does not affect | Does not affect |
| S383F | Increase | Does not affect | Does not affect |
| C396R | Does not affect | Does not affect | Does not affect |
| T404K | Does not affect | Does not affect | Does not affect |
| E430G | Does not affect | Does not affect | Does not affect |
| M432I | Does not affect | Does not affect | Does not affect |
| A436E | Does not affect | Does not affect | Does not affect |
| R441H | Increase | Does not affect | Does not affect |
| R471H | Does not affect | Does not affect | Does not affect |
| R471K | Does not affect | Does not affect | Does not affect |
| D473N | Does not affect | Does not affect | Does not affect |
| L474V | Does not affect | Does not affect | Does not affect |
| D479E | Does not affect | Does not affect | Does not affect |
| Q486K | Does not affect | Does not affect | Does not affect |
