# Supplementary material for: In silico analysis of the tryptophan hydroxylase 2 (TPH2) protein variants related to psychiatric disorders
Source: PLoS One. 2020 Mar 2;15(3):e0229730. doi: 10.1371/journal.pone.0229730 (PMC7051086; doi:10.1371/journal.pone.0229730)
Supplement: S3 Table — (DOCX) [file pone.0229730.s003.docx]

**S3 Table. Stability prediction of each TPH2 protein variant.**

| **Protein Variant** | **I-Mutant** | **FoldX** |
| --- | --- | --- |
| S22L | Does not affect | * |
| P25L | Decrease | * |
| G32D | Decrease | * |
| L36V | Decrease | * |
| L36P | Decrease | * |
| S41Y | Does not affect | * |
| S53R | Does not affect | * |
| R55C | Decrease | * |
| T64I | Does not affect | * |
| A65T | Decrease | * |
| V78I | Decrease | * |
| R82S | Decrease | * |
| L83V | Decrease | * |
| E86Q | Does not affect | * |
| R88H | Decrease | * |
| M91I | Decrease | * |
| R100Q | Decrease | * |
| R101Q | Decrease | * |
| Q124R | Does not affect | * |
| T134M | Does not affect | * |
| E145Q | Does not affect | * |
| R156Q | Decrease | Does not affect |
| P206S | Decrease | Decrease |
| R225Q | Decrease | Does not affect |
| R276S | Decrease | Decrease |
| P277L | Decrease | Decrease |
| R303W | Does not affect | Decrease |
| A328V | Does not affect | Decrease |
| I339M | Decrease | Does not affect |
| G345E | Does not affect | Decrease |
| D348H | Does not affect | Decrease |
| E363K | Does not affect | Decrease |
| A378T | Does not affect | Decrease |
| S383F | Does not affect | Does not affect |
| C396R | Does not affect | Increase |
| T404K | Decrease | Increase |
| E430G | Decrease | Decrease |
| M432I | Decrease | Decrease |
| A436E | Decrease | Decrease |
| R441H | Decrease | Decrease |
| R471H | Decrease | Decrease |
| R471K | Decrease | Does not affect |
| D473N | Decrease | Does not affect |
| L474V | Decrease | Decrease |
| D479E | Does not affect | Does not affect |
| Q486K | Does not affect | Does not affect |

*Unable to predict, since no structure has been experimentally determined for this region.
