# Supplementary figures and images for: In silico analysis of the tryptophan hydroxylase 2 (TPH2) protein variants related to psychiatric disorders
Source: PLoS One. 2020 Mar 2;15(3):e0229730. doi: 10.1371/journal.pone.0229730 (PMC7051086; doi:10.1371/journal.pone.0229730)

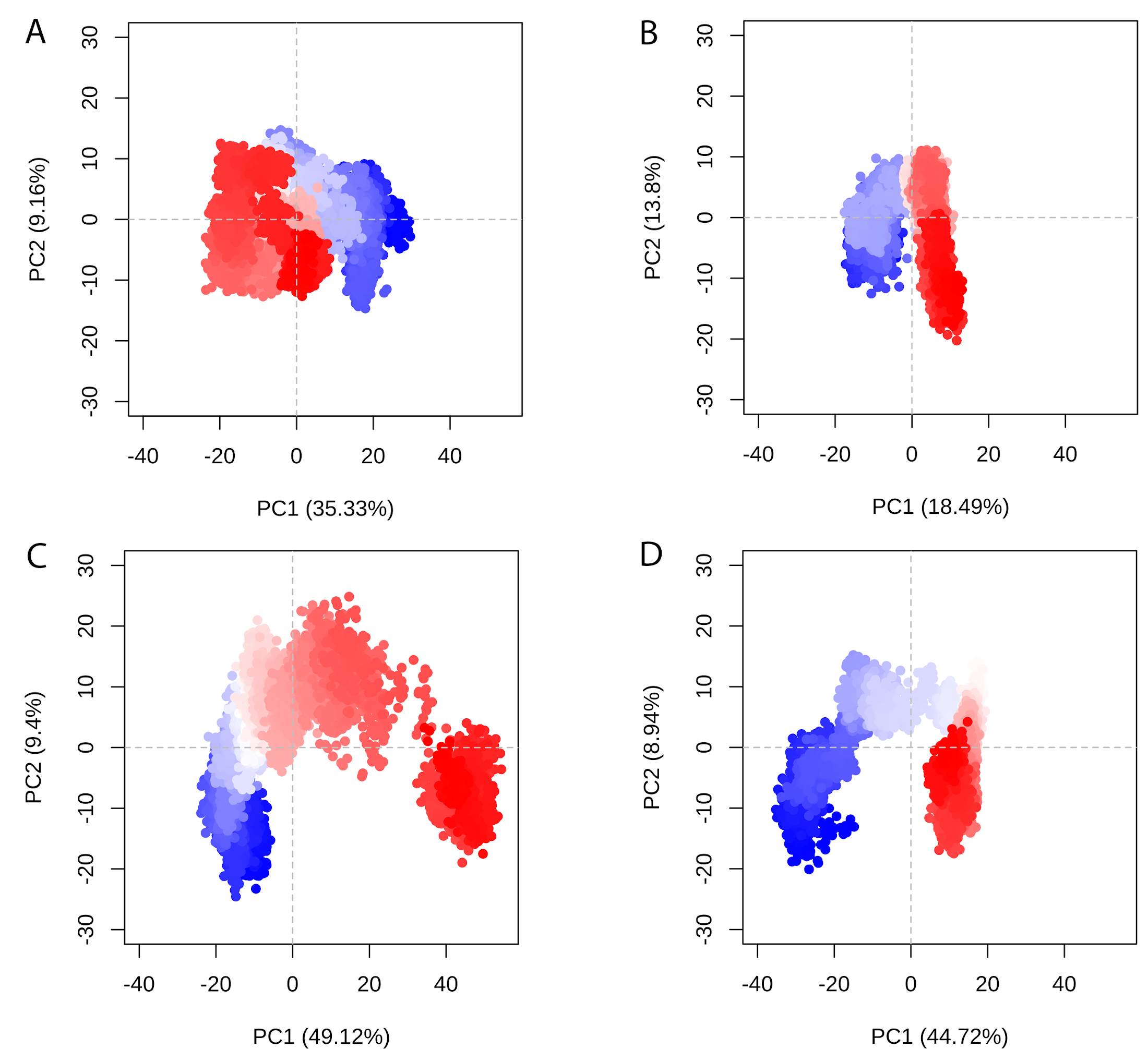

Supplement: S1 Fig — Projection of PC1 and PC2 extracted from the essential dynamics and the percentage of the total variance explained by them. The trajectory frames for TPH2 WT and variants are colored from blue to red according to time evolution. (A) PCA plot of WT TPH2. The first two PCs account for 44.49% of the total variance. (B) PCA plot of variant P206S. The first two PCs account for 32.29% of the total variance. (C) PCA plot of variant R303W. The first two PCs account for 58.52% of the total variance. (D) PCA plot of variant R441H. The first two PCs account for 53.66% of the total variance. (TIF) [file pone.0229730.s004.tif]

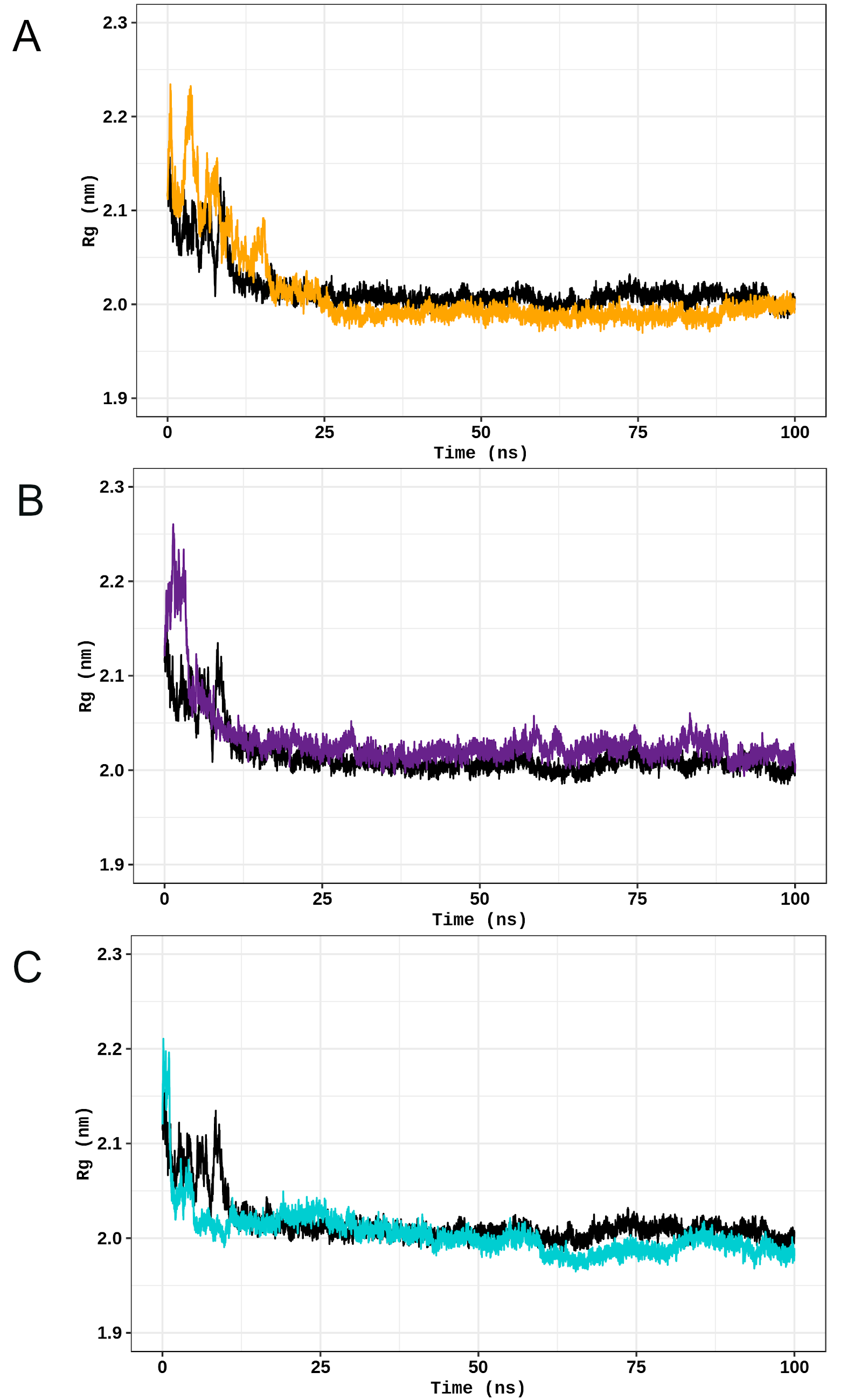

Supplement: S2 Fig — The Rg values of WT TPH2 and its variant at 300 K are shown as a function of time. (A) Comparison between the WT (black) and P206S variant (dark yellow). (B) Comparison between the WT (black) and R303W variants (purple). (C) Comparison between the WT (black) and R441H variant (turquoise). (TIF) [file pone.0229730.s005.tif]

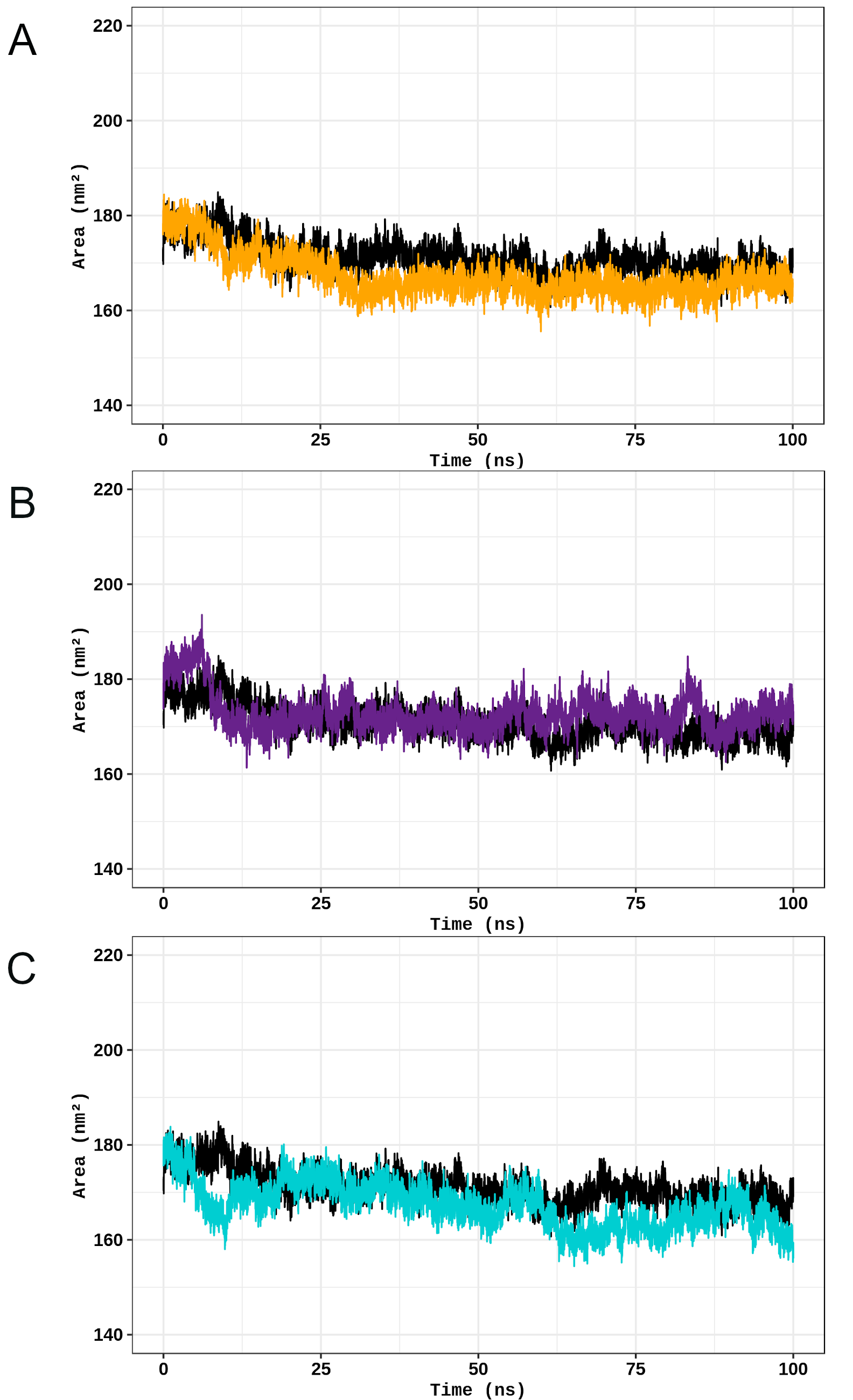

Supplement: S3 Fig — The SASA values of WT TPH2 and its variant at 300 K are shown as a function of time. (A) Comparison between the WT (black) and P206S variant (dark yellow). (B) Comparison between the WT (black) and R303W variant (purple). (C) Comparison between the WT (black) and R441H variant (turquoise). (TIF) [file pone.0229730.s006.tif]
